# Supplementary material for: Nanofiber-based platform for quantitative analysis of human oligodendrocyte ensheathment with pharmacological perturbations
Source: Stem Cell Reports. 2026 May 14;21(6):102923. doi: 10.1016/j.stemcr.2026.102923 (PMC13261957; doi:10.1016/j.stemcr.2026.102923)
Supplement: Document S1. Figures S1–S3 and Tables S1 and S2 [file mmc1.pdf]

Stem Cell Reports, Volume *21*

Supplemental Information

Nanofiber-based platform for quantitative analysis of human oligodendrocyte ensheathment with pharmacological perturbations

Satoshi Morita, Takayuki Kondo, Keiko Imamura, Yukako Sagara, Kayoko Tsukita, Hisanori Tokuda, Yoshihisa Kaneda, Takayuki Izumo, Yoshihiro Nakao, and Haruhisa Inoue

Figure S1

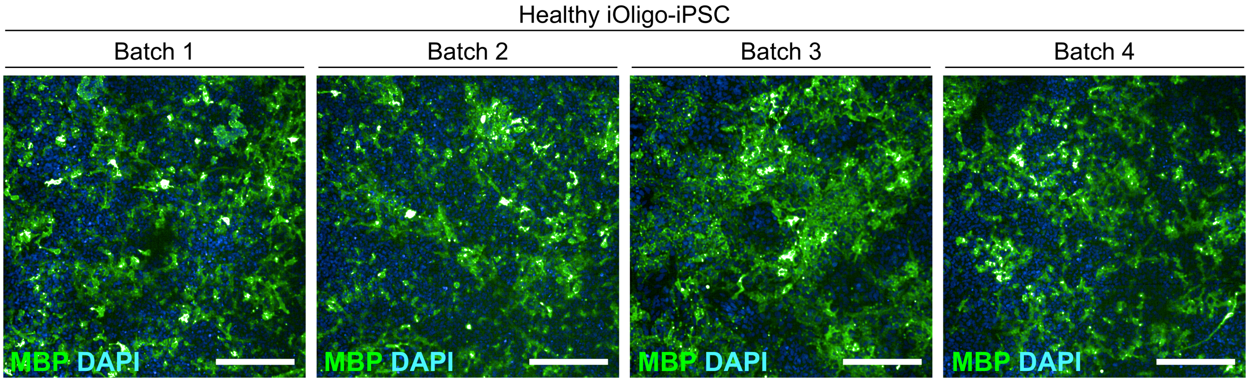
Healthy iOligo-iPSC
Batch 1
Batch 2
Batch 3
Batch 4
MBP DAPI
MBP DAPI
MBP DAPI
MBP DAPI

Figure S2

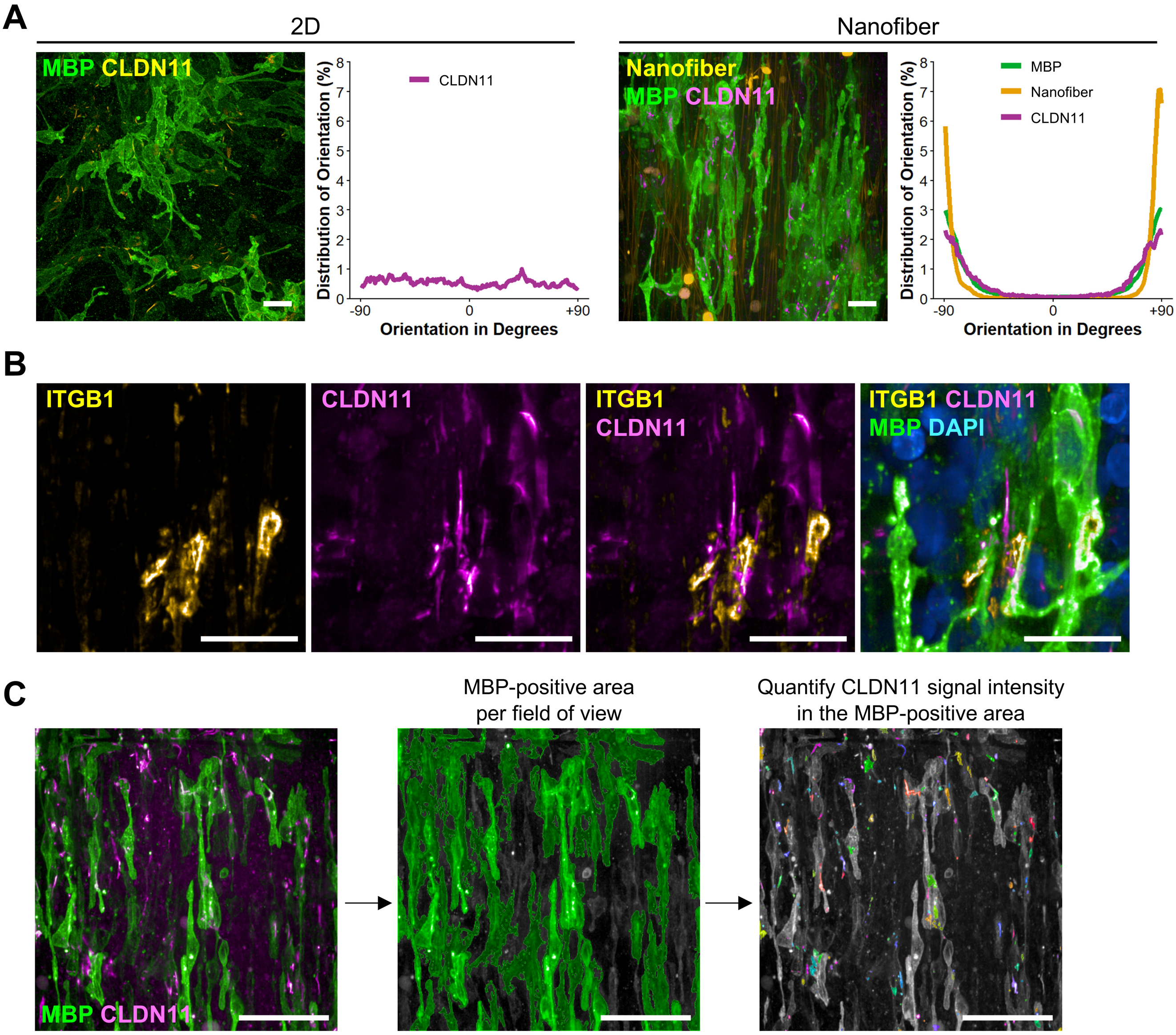

A
2D
Nanofiber
MBP CLDN11
Distribution of Orientation (%)
CLDN11
Orientation in Degrees
-90
0
+90
Nanofiber
MBP CLDN11
MBP
Nanofiber
CLDN11
B
ITGB1
CLDN11
ITGB1
CLDN11
ITGB1 CLDN11
MBP DAPI
C
MBP-positive area
per field of view
Quantify CLDN11 signal intensity
in the MBP-positive area
MBP CLDN11

Figure S3

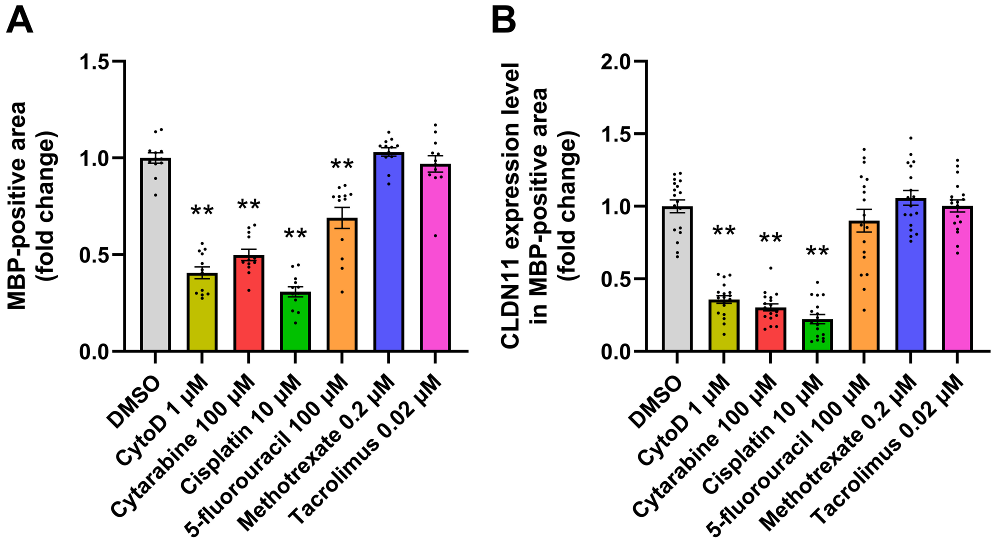
A
MBP-positive area
(fold change)
1.5
1.0
0.5
0.0
**
**
**
**
DMSO
CytoD 1 μM
Cytarabine 100 μM
Cisplatin 10 μM
5-fluorouracil 100 μM
Methotrexate 0.2 μM
Tacrolimus 0.02 μM
B
CLDN11 expression level
in MBP-positive area
(fold change)
2.0
1.5
1.0
0.5
0.0
**
**
**
DMSO
CytoD 1 μM
Cytarabine 100 μM
Cisplatin 10 μM
5-fluorouracil 100 μM
Methotrexate 0.2 μM
Tacrolimus 0.02 μM

Supplemental figure legends

Figure S1. Differentiated oligodendrocytes across independent batches.

Representative images of 201B7-iOligo from four independent differentiation batches cultured for 1 week with doxycycline. Scale bars, 200 μm.

Figure S2. Analysis of CLDN11 expression relative to nanofibers.

(A) Representative images of CLDN11 expression in conventional 2D culture and on nanofibers. The orientation of CLDN11, MBP, and nanofibers was quantified using the ImageJ OrientationJ plugin. Local orientation angles ranging from -90° to +90° were evaluated using the "Distribution" function based on the structure tensor method with a cubic spline gradient. Histograms show the normalized relative frequency (%) of the orientation distribution. Scale bars, 20 μm.

(B) Representative images of ITGB1 and CLDN11 expression in cells cultured for two weeks on nanofibers. Scale bars, 20 μm.

(C) Analytical workflow for MBP-positive area detection and quantification of CLDN11 signal intensity. Scale bars, 50 μm.

Figure S3. Evaluation of drug-induced leukoencephalopathy agents.

The MBP-positive area and CLDN11 signal intensity within the MBP-positive area were calculated. Data represent mean ± SE. n = 12 (A) or 18 (B) images were analyzed per condition, obtained from 3 independent cultures. Statistical comparisons were performed using Dunnett's test compared to DMSO control. $**p < 0.01$.

Table S1. The interbatch coefficient of variation, related to Figure 1.

Parameter	MBP-positive cells (%)
Batch 1	30.4
Batch 2	22.9
Batch 3	40.8
Batch 4	31.7
Mean ± SD	31.4 ± 7.3
CV (%)	23.2

Table S2. List of iPSCs used in this study

iPSCs	Age	Gender	Race	Disease
201B7	30s	Female	Caucasian	Healthy
HPS5652	30s	Male	Asian	Healthy
HPS1748	60s	Male	Japanese	Healthy
