# Supplementary material for: Nanofiber-based platform for quantitative analysis of human oligodendrocyte ensheathment with pharmacological perturbations
Source: Stem Cell Reports. 2026 May 14;21(6):102923. doi: 10.1016/j.stemcr.2026.102923 (PMC13261957; doi:10.1016/j.stemcr.2026.102923)
Supplement: Document S2. Article plus supplemental information [file mmc3.pdf]

STEM CELL REPORTS
Article
ISSCR
OPEN ACCESS

Nanofiber-based platform for quantitative analysis of human oligodendrocyte ensheathment with pharmacological perturbations

Satoshi Morita,[1,2,3] Takayuki Kondo,[1,2,4] Keiko Imamura,[1,2,4] Yukako Sagara,[2] Kayoko Tsukita,[1] Hisanori Tokuda,[3] Yoshihisa Kaneda,[3] Takayuki Izumo,[3] Yoshihiro Nakao,[3] and Haruhisa Inoue[1,2,4,5,*]
[1]Center for iPS Cell Research and Application (CiRA), Kyoto University, Kyoto, Japan
[2]iPSC-based Drug Discovery and Development Team, RIKEN BioResource Research Center (BRC), Kyoto, Japan
[3]Institute for Science of Life, Suntory Wellness Ltd., Kyoto, Japan
[4]Medical-risk Avoidance based on iPS Cells Team, RIKEN Center for Advanced Intelligence Project (AIP), Kyoto, Japan
[5]Lead contact
*Correspondence: haruhisa@cira.kyoto-u.ac.jp
https://doi.org/10.1016/j.stemcr.2026.102923

SUMMARY

Oligodendrocytes are glial cells responsible for myelination in the central nervous system, and their dysfunction underlies various neurological disorders. However, existing human oligodendrocyte models are limited by low efficiency and insufficient standardization. Here, we developed a well-defined system for differentiating human oligodendrocytes from induced pluripotent stem cells using transient transcription factor expression. To recapitulate key structural aspects of oligodendrocyte-axon interactions, we cultured oligodendrocytes on nanofibers mimicking axonal topology. On this platform, ultrastructural, live-imaging, and transcriptomic analyses demonstrated dynamic oligodendrocyte-nanofiber interactions and ensheathment-like wrapping. This process was accompanied by aligned CLDN11 expression along nanofibers, providing a quantifiable structural readout without overt maturation. To validate the utility of the platform, we quantified the effects of white matter toxins and pro-ensheathment lipids. Our findings establish a robust system for evaluating oligodendrocyte ensheathment modulators, particularly those affecting cytoskeletal organization and initial sheath formation, and for investigating the pathophysiology of human oligodendrocyte-related disorders.

INTRODUCTION

Oligodendrocytes, central nervous system (CNS) glial cells that form myelin, are essential for efficient axonal transmission (Bercury and Macklin, 2015) and controlling brain plasticity (de Faria et al., 2021). Myelination begins during brain development in the third trimester of pregnancy and steadily increases throughout childhood (Hasegawa et al., 1992). In the adult brain, myelination is dynamically regulated by neural activity. Oligodendrocyte dysfunction is observed during aging (Gong et al., 2023) and across multiple pathological conditions, including neurodegenerative diseases (Depp et al., 2023; Nasrabady et al., 2018), multiple sclerosis (MS) (Lubetzki et al., 2020), white matter dystrophies (Gencic et al., 1989), and drug-induced leukoencephalopathies (Dietrich et al., 2006; Han et al., 2008), highlighting oligodendrocytes as important therapeutic targets. Mature myelinating oligodendrocytes differentiate from stem cells via progenitor cells (Jäkel et al., 2019). Multiple transcription factors including SOX10, OLIG2, and NKX6-2 have been identified as key regulators of this differentiation process (Neman and de Vellis, 2008). Forced expression of these factors accelerates differentiation, overcoming conventional challenges such as long culture times and low efficiency.

Most oligodendrocyte studies rely on rodent models. However, human white matter is structurally larger, exhibiting significant differences in oligodendrocyte gene and protein expression compared to rodents (Gargareta et al., 2022; Jorstad et al., 2023; Ransom et al., 2021). Another difference is developmental timing, with myelination continuing until approximately the age of 20 years in humans, whereas major myelination is completed within a few weeks after birth in rodents (Zeiss, 2021). Additionally, important species differences exist in disease models; MS, a human-specific disease, makes it difficult to obtain translatable insights from rodent models (Ransohoff, 2012). These facts highlight the importance of human oligodendrocytes for disease research. Due to these species differences, therapeutic candidates that show promising results in rodent models frequently prove ineffective in human clinical trials, thus creating a significant barrier to the development of new treatments.

Neural cells differentiated from human induced pluripotent stem cells (iPSCs) are representative models for recapitulating the human brain and spinal cords in culture dishes (Inoue et al., 2014). However, although iPSC technology has enabled the use of human oligodendrocytes (García-León et al., 2018; Madhavan et al., 2018), robust models for evaluating human oligodendrocyte ensheathment capacity are still lacking. Specifically, there is a need for an assay system suitable for quantitative evaluation that can recapitulate the critical physical structure of axons while avoiding the complexities of neuron co-culture systems. An ideal platform for drug screening applications, therefore, requires a standardized workflow with high

Published by Elsevier Inc. on behalf of International Society for Stem Cell Research.
This is an open access article under the CC BY license (http://creativecommons.org/licenses/by/4.0/).

differentiation efficiency, rapid evaluation timelines, and objective, quantitative readouts. Developing an *in vitro* platform to study the interactions between neurons and oligodendrocytes, particularly the initial process of axonal ensheathment, is essential for evaluating oligodendrocyte dysfunction in neuropsychiatric and neurodegenerative diseases. Thus, we attempted to address these issues and establish a model in order to understand oligodendrocyte-related disorders and develop compound screening assays targeting ensheathment and structural organization.

RESULTS

Doxycycline-dependent induction of oligodendrocyte differentiation

Oligodendrocytes can differentiate from iOligo-iPSCs by expressing three transcription factors: SOX10, OLIG2, and NKX6-2 (Ehrlich et al., 2017). To avoid variable lentiviral infection efficiency, we created iOligo-iPSC clones expressing these factors in a doxycycline-dependent manner via the piggyBac system (Kim et al., 2015). Healthy human iOligo-iPSCs were differentiated into neural progenitor cells (NPCs), which were then treated with doxycycline for 1 week to induce expression of the three transcription factors (Figure 1A). We confirmed that the expression of the three induced transcription factors, SOX10, OLIG2, and NKX6-2, was upregulated in a doxycycline concentration-dependent manner (Figure 1B). PDGFR-α, which is specifically expressed in oligodendrocyte progenitor cells, showed a marginally decreased expression (Figure 1C). Additionally, the expression of mature oligodendrocyte marker genes such as MBP, MYRF, PLP1, CLDN11, and PMP2 increased (Figure 1D). One week after doxycycline addition, the expression of MBP, a mature oligodendrocyte marker protein, increased, and approximately 30% of the cells were MBP-positive (Figures 1E; Table S1). These results demonstrate that cells expressing mature oligodendrocyte markers can be obtained with high efficiency over a short period of time. Similar results were obtained in other healthy iPSC lines, confirming that the induction of differentiation was possible regardless of the iPSC line (Figures 1F and 1G). Furthermore, consistent results were obtained across different differentiation batches, with an inter-batch coefficient of variation of 23.2% (Figure S1; Table S1).

Co-culture of oligodendrocytes and neurons

To investigate the interaction between oligodendrocytes and neurons, oligodendrocytes were co-cultured with neurons differentiated from the same healthy human iPSC line (Figure 2A). Oligodendrocytes were observed ensheathing the neuronal processes (Figure 2B).

Process extension of oligodendrocytes differentiated on nanofibers

Oligodendrocytes were differentiated on nanofibers composed of polycaprolactone fibers and arranged unidirectionally to mimic the axonal alignment found in white matter (Figure 3A). The fiber diameter was 0.7 μm, which was near the thickness of actual neuronal axons (von der Bey et al., 2023; Lee et al., 2012). NPCs were cultured on the nanofibers for 2 weeks and differentiated into oligodendrocytes. After 2 weeks, oligodendrocytes extended processes of various lengths and branches along the nanofibers (Figure 3B), with morphologies indicating fiber wrapping (Figure 3C). Cross-sectional analysis using transmission electron microscopy (TEM) visualized the interaction between cells and nanofibers. Notably, we identified structures where cells appeared to ensheath the nanofibers (Figure 3D). Additionally, live imaging was performed using an MBP-tdTomato iPSC line (Takagi et al., 2024). We performed time-lapse imaging for 48 h to observe how oligodendrocytes interact with these process-like structures in real time. Oligodendrocyte processes were observed repeatedly extending and retracting along the nanofibers (Figures 3E; Video S1). This dynamic movement is thought to represent a probing behavior, where the cell actively searches the nanofiber for appropriate cues for initiating ensheathment. This exploratory phase likely precedes the eventual stabilization and structural organization of the sheath around nanofibers.

Transcriptomic characteristics of oligodendrocytes cultured on nanofibers

To elucidate the molecular mechanisms underlying the enhanced ensheathment phenotype observed on nanofibers, we performed RNA-seq analysis comparing oligodendrocytes differentiated in conventional 2D cultures with those cultured on nanofibers. Principal component analysis and volcano plots confirmed distinct transcriptomic profiles and identified differentially expressed genes (DEGs) (Figures 4A and 4B). Gene ontology (GO) enrichment analysis revealed a global functional shift: categories related to "ECM organization," "lipid metabolism," and "cell adhesion and wound healing" were significantly upregulated, whereas "axonogenesis" was downregulated (Figure 4C). Notably, although the expression of mature oligodendrocyte markers remained unchanged and these genes were not identified as DEGs (Figure 4E), we observed a significant upregulation of genes involved in cell adhesion, lipid synthesis and transport, and extracellular matrix (ECM) organization. Conversely, genes associated with axonal elongation were downregulated (Figure 4D). These data suggest that the nanofiber environment does not upregulate oligodendrocyte maturation markers but rather suppresses exploratory process elongation while promoting adhesive wrapping and lipid metabolism,

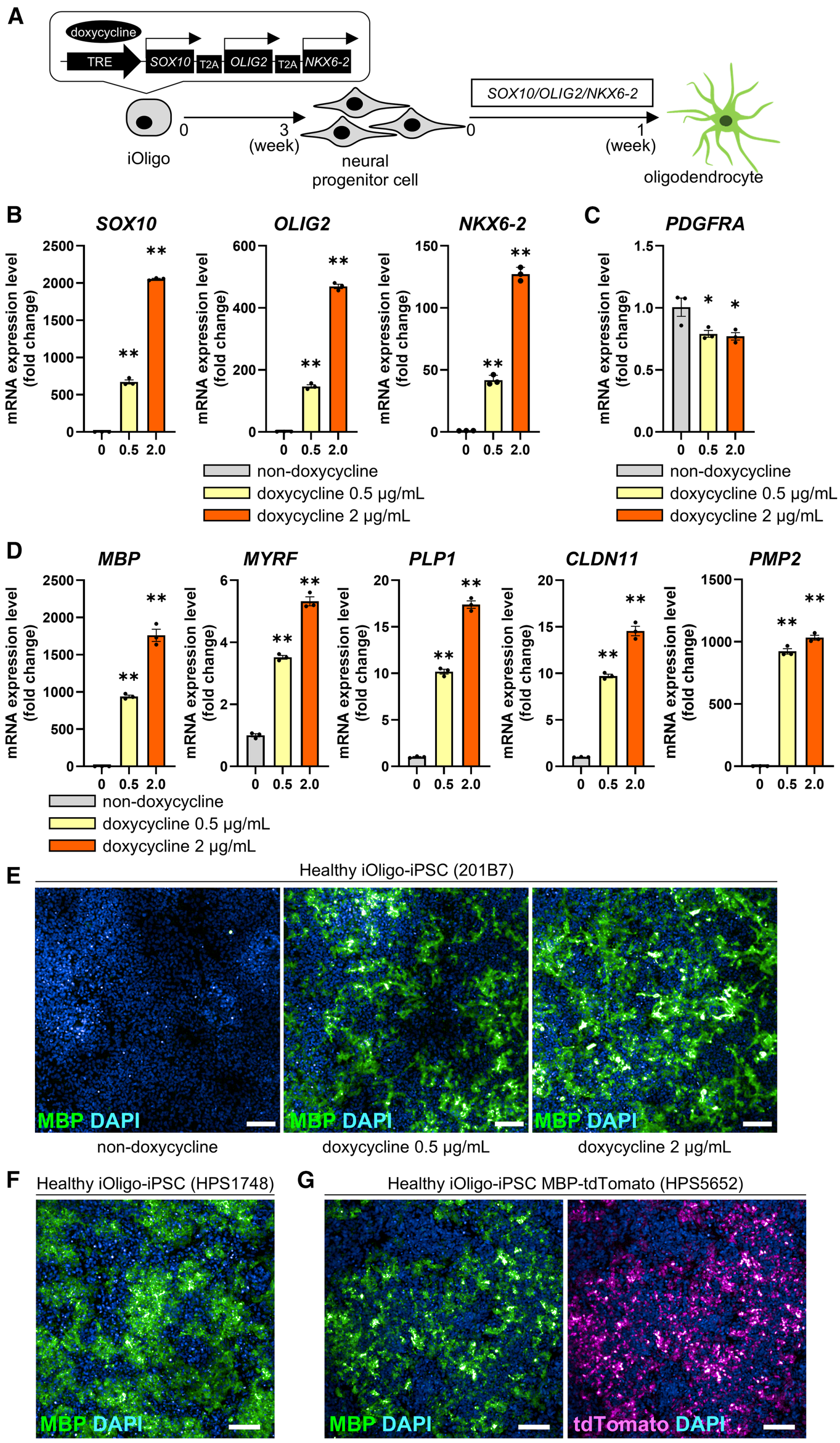

Figure 1. Doxycycline-dependent induction of oligodendrocyte differentiation

(A) Schematic diagram of conditions used to induce human iPSC-derived oligodendrocytes.

(B–D) mRNA expression levels of 201B7-iOligo cultured for 1 week with doxycycline. Data represent the mean ± SE, $n = 3$ independent cultures per condition. Statistical comparisons were performed using Dunnett's test compared to DMSO control, $*p < 0.05$, $**p < 0.01$.

(E–G) Representative images of 201B7-iOligo (E), HPS1748-iOligo (F), and HPS5652-iOligo (G) cultured for 1 week with doxycycline. Scale bars, 100 μm.

thereby priming the cells for ensheathment and structural organization. To investigate the molecular mechanism underlying the interaction with nanofibers, we focused on integrin signaling. Given that the nanofibers used in this study were coated with laminin, we specifically examined the expression of ITGA6 and ITGB1, which are known to recognize extracellular laminin and signal to promote oligodendrocyte ensheathment (Buttery and

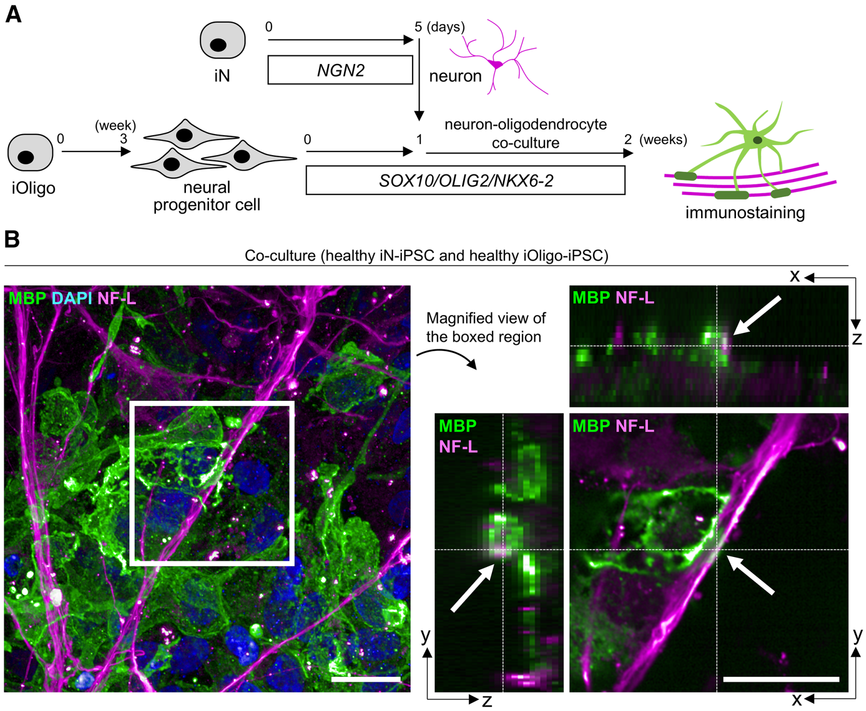

Figure 2. Co-culture of oligodendrocytes and neurons

(A) Schematic diagram of the neuron-oligodendrocyte co-culture conditions.

(B) Representative images of 201B7-iN and 201B7-iOligo co-cultured for 1 week with doxycycline. The white frame area with neuron-oligodendrocyte interactions is extended to the right picture. Scale bars, 20 μm.

ffrench-Constant, 1999). Our data confirmed that while ITGA6 and ITGB1 were not identified as DEGs, they were constitutively expressed in this system (Figure 4F). This suggests that oligodendrocytes have the necessary machinery to sense and respond to the laminin-coated nanofibers, potentially triggering downstream cytoskeletal reorganization (O'Meara et al., 2011).

Establishment of a compound screening system using CLDN11 expression

Adhesion molecules are expressed during axonal ensheathment. CLDN11 is a representative adhesion molecule specifically expressed in myelin (Gjervan et al., 2024; Gow et al., 1999) that enhances the insulating properties of myelin (Devaux and Gow, 2008). First, NPCs were differentiated into oligodendrocytes on nanofibers for 2 weeks. CLDN11 was expressed along the nanofibers, as confirmed by immunostaining (Figures 5A, 5D, and 5G). The observed expression pattern of CLDN11 suggests that oligodendrocyte processes are wrapping around the fibers. The orientation distribution of CLDN11 aligns with the direction of the nanofibers, whereas no preferential orientation is observed in the absence of nanofibers (Figure S2A). Similarly, the orientation of the cytoplasm, visualized by MBP staining, also showed alignment with the nanofibers, indicating that the physical cue orients the entire cell. To further investigate the molecular spatial relationship, we performed immunostaining for ITGB1 and CLDN11. Aligned CLDN11 signals were observed in close proximity to ITGB1 signals (Figure S2B). This spatial arrangement suggests that CLDN11 alignment is organized near sites of integrin-mediated adhesion, representing an initial phase of structural organization required for ensheathment. We constructed a system to evaluate the effects of compounds on oligodendrocyte ensheathment and structural organization by immunostaining and following image analysis for MBP and CLDN11 (Figure S2C). After oligodendrocytes were differentiated on the nanofibers, various compounds were added to the culture medium and image analysis was performed. Compounds that modulate oligodendrocyte ensheathment and myelination have been identified by the analysis of rodent-derived oligodendrocytes (Bernardo et al., 2017; Nawaz et al., 2015; Schepers et al., 2023; Trapp and Bernsohn, 1978). As a proof-of-concept to validate our evaluation system, we assessed its performance by using known ensheathment modulators with different mechanisms of action. When CytoD (cytochalasin-D), a known inhibitor of ensheathment, was added, decreased CLDN11 expression levels were observed (Figures 5A–5C). Conversely, when roflumilast, a phosphodiesterase (PDE) inhibitor reported to promote ensheathment formation, was added, the CLDN11 expression increased (Figures 5D–5F). Moreover, the addition of docosahexaenoic acid (DHA) and arachidonic acid (ARA), which are polyunsaturated fatty acids essential for myelin structure development, resulted in increased CLDN11 expression (Figures 5G–5I). To further

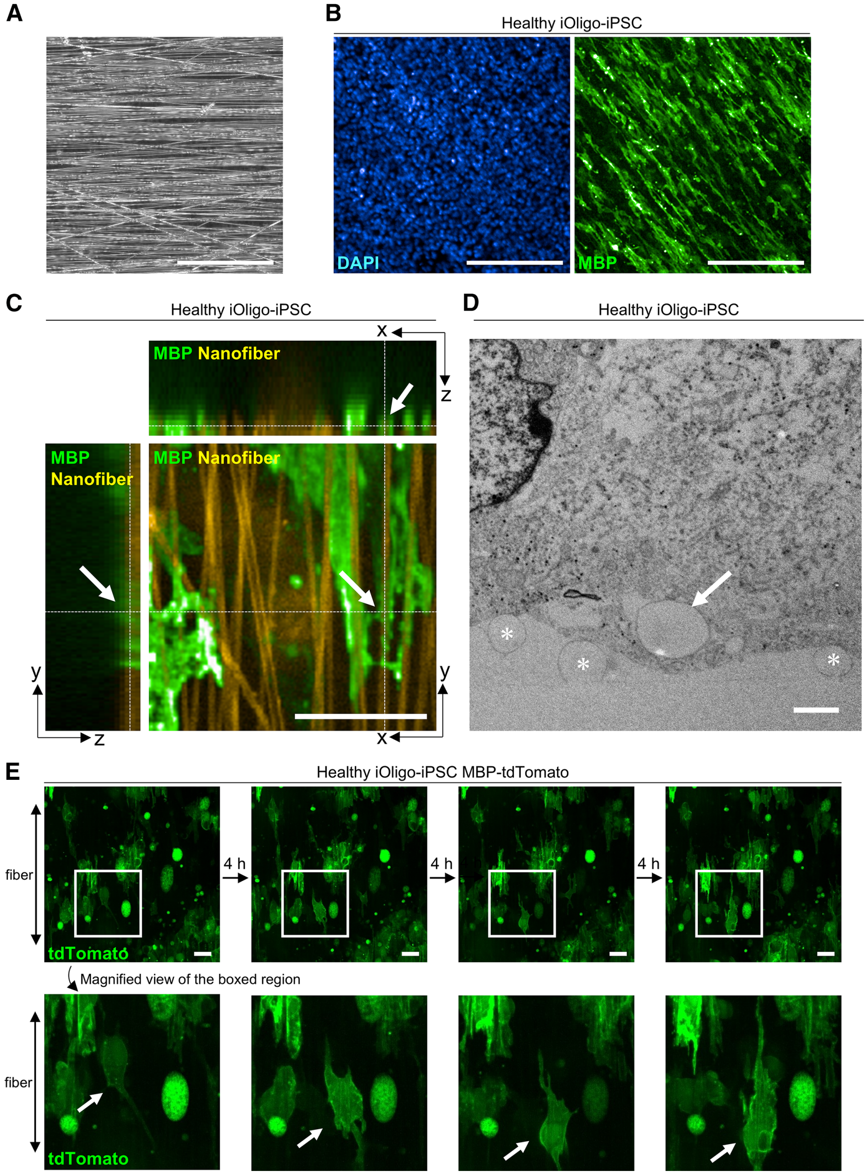

Figure 3. Process extension of oligodendrocyte differentiated on nanofibers

(A) Representative bright-field images of the nanofibers used in this study. Scale bars, 200 μm.

(B) Representative images of MBP-positive 201B7-iOligo cells cultured for 2 weeks on nanofibers. Scale bars, 200 μm.

(C) Extended image of MBP-positive 201B7-iOligo cells cultured for 2 weeks on nanofibers. Yellow lines show the nanofibers coated with FITC-labeled poly-L-lysine. Scale bars, 20 μm.

(D) Transmission electron microscopy images of cross-sections of fixed cells cultured on nanofibers. White arrows indicate cross-sections of nanofibers ensheathed by cells, while asterisks mark nanofibers without ensheathment. Scale bars, 1 μm.

(E) Representative time-lapsed images of differentiated oligodendrocytes using iOligo-iPSC expressing tdTomato at the MBP C-terminus. Scale bars, 20 μm.

validate the utility of our platform for toxicological screening, we assessed the effects of drugs known to cause leukoencephalopathy clinically. We selected cytarabine, cisplatin, 5-fluorouracil (5-FU), methotrexate, and tacrolimus, and treated the cells at concentrations approximating the maximum blood concentration (Cmax) reported in clinical pharmacokinetic data. Cytarabine and cisplatin significantly reduced the CLDN11 expression, similar to the effects observed with CytoD. In contrast, 5-FU, methotrexate, and tacrolimus showed no significant changes in CLDN11 expression (Figures S3A and S3B). These results suggest that our platform is sensitive to specific types of drug-induced oligodendrocyte toxicity and can serve as a useful tool for screening compounds with potential risks of the early event of white matter damage. Using oriented CLDN11 expression as an indicator, ensheathment capacity and toxicities on oligodendrocytes can be evaluated, thereby establishing a system capable of assessing diverse compounds.

DISCUSSION

We established a robust induction of human oligodendrocyte differentiation via a combination of transcription factor expressions using TetON technology and nanofiber

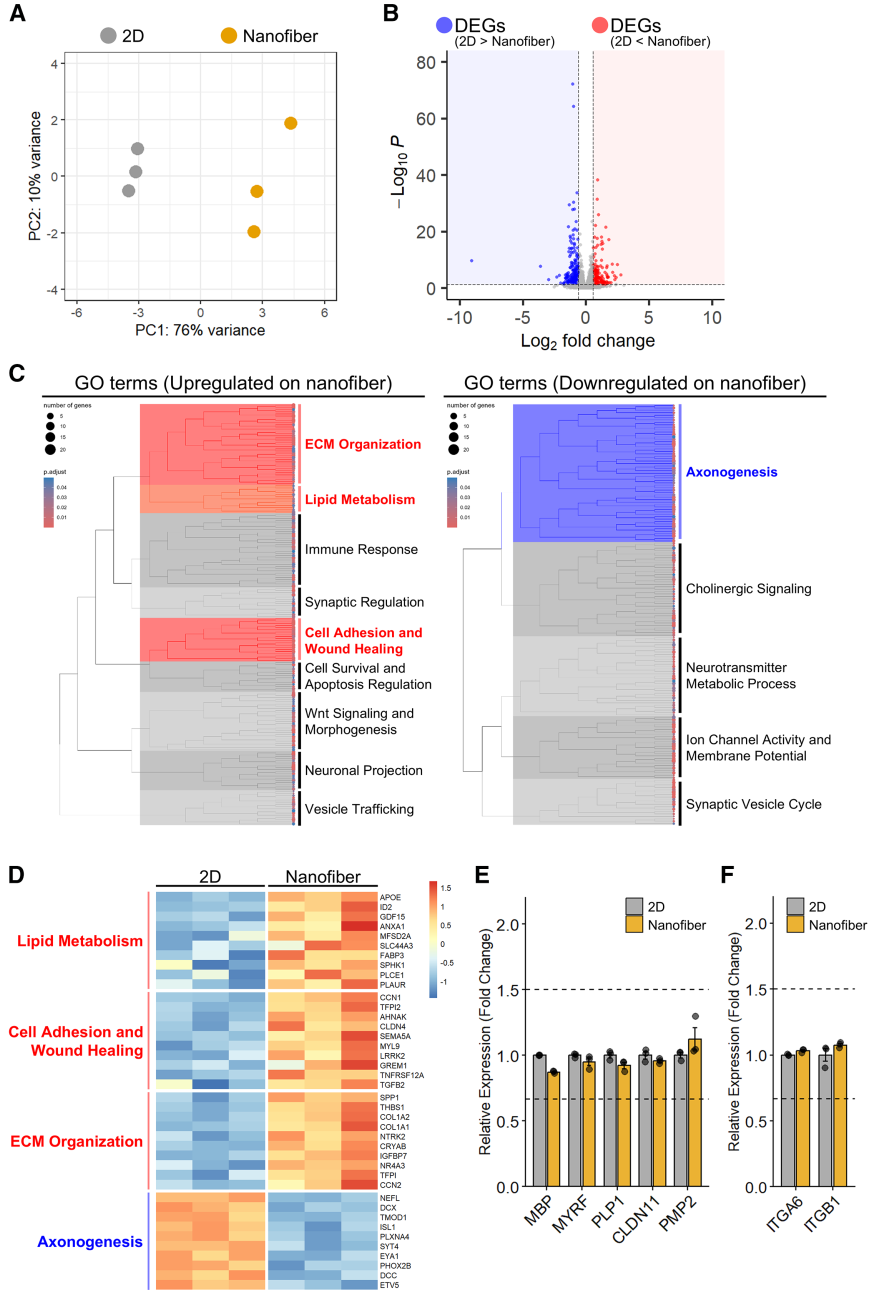

Figure 4. Transcriptomic profiling reveals a functional shift toward a promyelinating state when cultured on nanofibers

(A) Principal component analysis (PCA) plot showing distinct clustering between the 2D and nanofiber groups (n = 3 independent cultures per condition).

(B) Volcano plot displaying differentially expressed genes (DEGs). Red dots indicate upregulated DEGs, and blue dots indicate downregulated DEGs in the nanofiber group (adjusted p value < 0.05, |FoldChange| > 1.5).

(C) Functional clustering of significantly enriched GO terms. Representative clusters for upregulated (ECM organization, lipid metabolism, cell adhesion, and wound healing) and downregulated (axonogenesis) processes are highlighted.

(D) Heatmap of the top 10 representative DEGs for the selected functional categories (ECM organization, lipid metabolism, cell adhesion and wound healing, and axonogenesis). Genes were selected based on the lowest adjusted p value within each category. Overlapping genes were assigned to a single category based on their canonical functions to avoid redundancy. The color scale represents the Z score of normalized counts.

(E and F) Expression of mature oligodendrocyte markers (E) and laminin receptors (F). Data represent mean ± SE (n = 3 independent cultures per condition). The dashed lines indicate a |FoldChange| of 1.5.

culture. Ultrastructural, live-imaging, and transcriptomic analyses demonstrated dynamic oligodendrocyte-nanofiber interactions characterized by exploratory process behavior and ensheathment-like wrapping. By employing the oriented expression of CLDN11 as a quantitative indicator of structural organization, we successfully developed a platform to evaluate the initial phase of sheath formation, specifically axonal ensheathment, transforming a standard differentiation protocol into a versatile tool for compound screening.

Improved reproducibility and time reduction in oligodendrocyte differentiation

Recent studies have reported improvements in the efficiency of differentiation induction of human iPSC-derived oligodendrocytes. Following the report of a differentiation-induction method using SOX10, OLIG2, and NKX6-2 (Ehrlich et al., 2017), subsequent studies demonstrated that mature oligodendrocytes could be obtained with SOX10 alone (García-León et al., 2018). Additionally, a differentiation induction method using SOX9 alone was

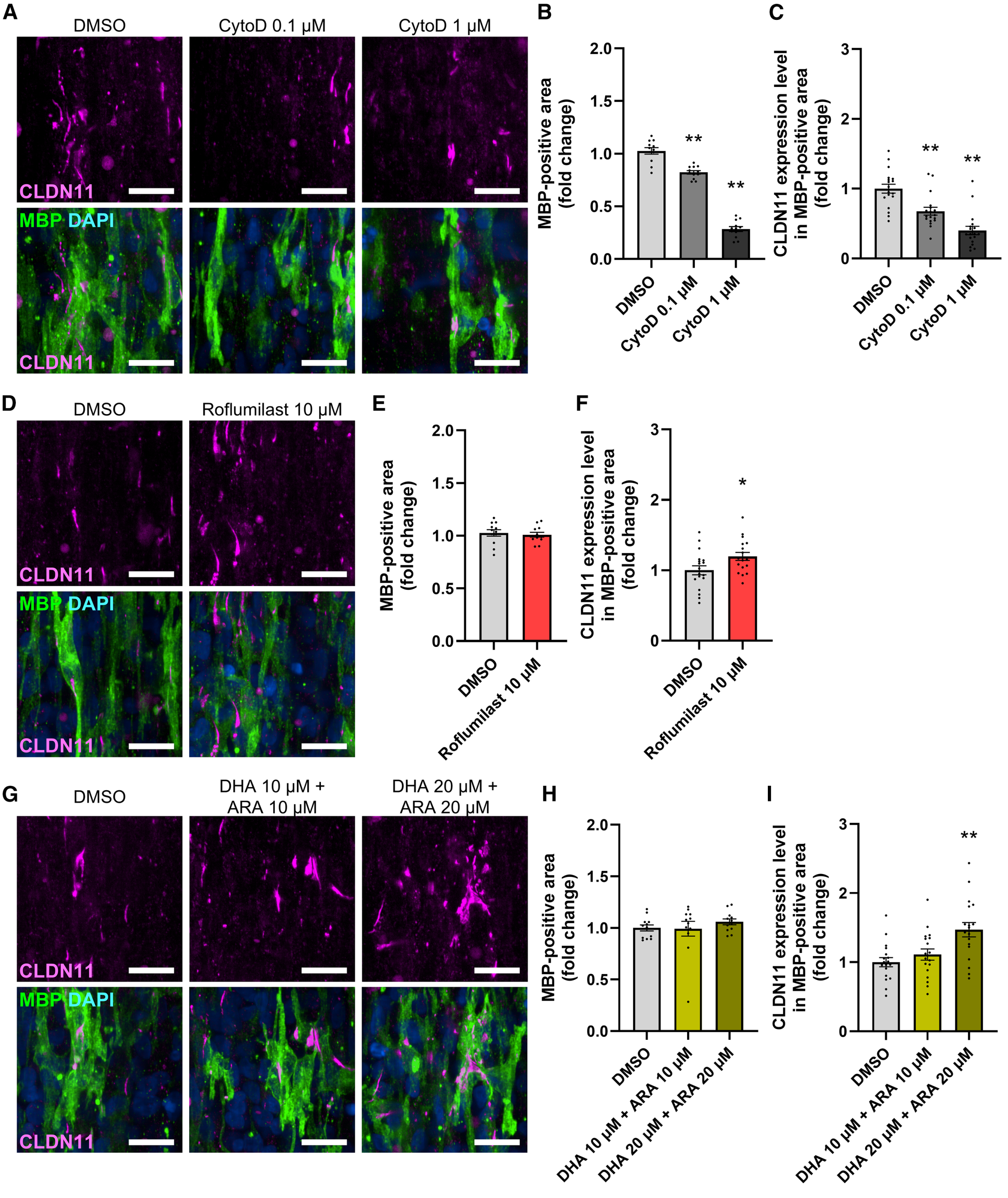

Figure 5. Establishment of a compound screening system using claudin-11 expression

201B7-iOligo was differentiated on nanofibers for 1 week. CytoD (A–C), roflumilast (D–F), and DHA and ARA (G–I) were added to the medium and cultured for an additional week. (A, D, G) Representative immunostained images of MBP and CLDN11.
(B, C, E, F, H, I) Results of subsequent image analysis. The MBP-positive area and CLDN11 signal intensity within the MBP-positive area were calculated. Data represent mean ± SE. $n = 12$ (B, E, H) or 18 (C, F, I) images were analyzed per condition, obtained from 3 independent cultures. Statistical comparisons were performed using unpaired t test (F) or Dunnett's test compared to DMSO control (B, C, I). *$p < 0.05$, **$p < 0.01$. Scale bars, 20 µm.

demonstrated (Ng et al., 2021). Thus, the introduction of transcription factors can significantly shorten the period of oligodendrocyte generation. In our study, approximately 30% of cells were MBP-positive after 1 week of transcription factor induction, and the use of TetON technology improved the stability of differentiation induction,

yielding robust reproducibility across different iPSC lines. For compound evaluation, the stability of differentiation induction and reproducibility across different cell lines are important, and this approach is expected to be utilized as a foundational technology for elucidating the pathologies of oligodendrocyte-related disorders and developing therapeutic approaches.

Species differences between humans and mice

Highlighting the need for human models to bridge the translational gap, recent proteomic analyses show significant species-specific differences between human and mouse oligodendrocytes (Gargareta et al., 2022). Peripheral myelin protein 2 (PMP2) was reported to be a representative factor specifically expressed in human but not mouse oligodendrocytes. We confirmed the PMP2 expression in the differentiated human oligodendrocytes, which suggests the importance of human model systems in the translation from preclinical research to clinical applications.

Physiological significance of the nanofiber system

Models of oligodendrocyte ensheathment using nanofibers represent a technology that has advanced rapidly in recent years. Fibers require a diameter of 0.4 μm or greater for oligodendrocyte membrane wrapping (Lee et al., 2012). Our 0.7 μm fibers exceeded this required thickness, providing a microenvironment mimicking neuronal axons. The complexity of the scaffolds influences the cellular responses. Compared to conventional 2D systems, microscale three-dimensional environments can reproduce complex cell-to-cell or cell-extracellular matrix interactions and establish platforms that more closely resemble the *in vivo* microenvironment (Nakano et al., 2023). Our transcriptomic analysis revealed that culturing on nanofibers significantly upregulated genes related to lipid metabolism, cell adhesion, and ECM organization, while downregulating axonogenesis-related genes. Lipid synthesis is essential for the massive membrane expansion required for ensheathment and subsequent myelination (Nave and Werner, 2021). Although TEM analysis in our study revealed that oligodendrocytes ensheathed the nanofibers, compact multi-layered myelin lamellae were not observed. However, the dynamic extending and retracting movements observed in time-lapse imaging, combined with the transcriptomic shift toward lipid synthesis, strongly suggest that the oligodendrocytes on nanofibers are in an active state of ensheathment and pro-myelinating molecular state. Collectively, these findings suggest that our nanofiber system successfully recapitulates the critical initial phase of myelination (Sherman and Brophy, 2005), where cells recognize the physical cue, synthesize necessary lipids, and initiate membrane wrapping, making it a highly suitable model for screening compounds that modulate these specific processes. Mechanistically, the interaction between laminin-coated nanofibers and cells is likely mediated by specific receptors. We confirmed the constitutive expression of ITGA6 and ITGB1, which are known to promote laminin-mediated ensheathment (Buttery and ffrench-Constant, 1999). Furthermore, immunostaining revealed that aligned CLDN11 signals localize in close proximity to ITGB1. This suggests a hierarchical model where integrin-mediated adhesion to the laminin scaffold spatially guides the downstream structural alignment of CLDN11, thereby organizing the membrane layers for ensheathment. This interaction likely triggers the downstream upregulation of genes driving cell adhesion, lipid synthesis, and ECM organization observed in our study.

Clinical significance of CLDN11 expression

In our study, the aligned expression of CLDN11 along the nanofibers suggests the formation of polarized structural organization. CLDN11 is one of the myelin-specific adhesion factors that has been shown to be essential for proper nerve conduction. In CLDN11-deficient mice, nerve conduction is reduced by 60% (Devaux and Gow, 2008). This supports the understanding that CLDN11 expression levels serve as a quantitative readout for oligodendrocyte ensheathment. The clinical relevance of CLDN11 is also well established. Mutations in CLDN11 have been identified as the cause of hypomyelinating leukodystrophy 22 (HLD22) (Riedhammer et al., 2021). Consistent with this, CLDN11 expression is significantly decreased in MS lesions (Uchida et al., 2019), supporting its value as an indicator in pathological research. Furthermore, we observed a population of MBP-positive cells with low CLDN11 expression in our system. We interpreted these cells as being immature oligodendrocytes that are committed to the lineage but have not yet achieved the structural organization required for myelination. This distinction highlights the value of CLDN11 as a specific marker for structural organization, distinct from differentiation markers such as MBP. Myelination is a multistep process involving initial axonal contact and ensheathment, followed by membrane compaction to form the mature insulating sheath (Sherman and Brophy, 2005). While we successfully observed robust axon ensheathment on the nanofibers, our TEM analysis indicated that these structures had not yet reached a fully compacted stage characterized by major dense lines. However, it is important to note that the initial ensheathment itself is a critical developmental milestone that provides metabolic support to axons, distinct from the electrical insulation provided by compaction (Nave and Werner, 2021). Furthermore, quantitative assessment of ensheathment efficiency in engineered *in vitro* models has been established as a valid metric for evaluating oligodendrocyte responses to external cues (Yang et al., 2025). To rigorously evaluate

this process, we distinguished between membrane expansion and structural organization by assessing both MBP and CLDN11. While MBP expression reflects the extent of membrane expansion, CLDN11 is a critical component of autotypic tight junctions, which constitute the radial component of CNS myelin (Gjervan et al., 2024). The specific linear alignment of CLDN11 observed in our system suggests the initiation of molecular organization and polarization required for myelin assembly (Morita et al., 1999). Therefore, we define CLDN11 distribution as a sensitive index of "structural alignment," providing a qualitative evaluation that complements the quantitative MBP-based ensheathment metrics.

Validation of the model using reported compounds

In our assay system, the effects of compounds reported to modulate myelination (CytoD, Roflumilast, DHA, and ARA) showed the same direction of efficacy as those reported in previous studies using rodent-derived oligodendrocytes. First, treatment with CytoD resulted in a decrease in both CLDN11 and MBP signals. While the previous report described the morphological disruption of the actin cytoskeleton by CytoD (Nawaz et al., 2015), our results suggest that this cytoskeletal collapse leads to process retraction and reduction in the MBP-positive area. On the other hand, treatment with roflumilast, DHA, and ARA significantly increased the oriented expression of CLDN11 along nanofibers, suggesting enhanced ensheathment capacity. The effects of roflumilast, a PDE inhibitor, support a mechanism of ensheathment via the cAMP pathway, while the effects of fatty acids support the importance of lipid metabolism. Reduced maternal intake of polyunsaturated fatty acids from pregnancy through lactation causes abnormalities in brain myelination, and DHA promotes the differentiation of rat-derived oligodendrocytes, which is consistent with our results (Bernardo et al., 2017; Trapp and Bernsohn, 1978). However, total MBP expression levels remained unchanged in our results. This finding differs from previous studies reporting that these compounds increased MBP expression. For instance, Schepers et al. reported that roflumilast promotes myelination in rat Schwann cells, and Bernardo et al. showed that DHA increases MBP expression in rat OPCs (Bernardo et al., 2017; Schepers et al., 2023). These discrepancies are likely attributable to differences in species, cell types, and most critically the maturation stage. While previous studies focused on Schwann cells in the peripheral nervous system or the differentiation phase of OPCs in rodents, the present study evaluates human CNS oligodendrocytes that have already differentiated and express high levels of MBP. Therefore, the observed effects in our study likely reflect the promotion of structural organization and membrane ensheathment in committed oligodendrocytes rather than the induction of *de novo* differentiation. Consistent with this interpretation, our transcriptomic data showed that nanofiber culture promotes lipid metabolism essential for membrane expansion without altering differentiation marker levels. These results suggest that using oriented CLDN11 expression as an indicator allows for the sensitive detection of structural organization, specifically the transition to the ensheathment phase, even when the total quantity of oligodendrocyte differentiation markers remains stable.

Utility of the compound assay system

Our system successfully reproduced the beneficial effects of these compounds on structural organization and demonstrated that a high-throughput evaluation of compounds is possible, confirming its utility in compound screening. Furthermore, this system is applicable not only for discovering ensheathment-promoting compounds but also for the toxicological screening of candidate compounds. In this study, we successfully detected the toxicity of cytarabine and cisplatin, which are clinically known to induce leukoencephalopathy. Interestingly, while cytarabine- and cisplatin-induced morphological changes similar to those observed with CytoD, no significant changes were detected with 5-FU, methotrexate, or tacrolimus, which also carry a risk of leukoencephalopathy. This discrepancy likely reflects differences in the underlying pathological mechanisms of white matter injury. Cytarabine and cisplatin are known to exert direct cytotoxicity on oligodendrocytes. Previous studies have demonstrated that these agents induce rapid apoptosis in both oligodendrocyte progenitor cells and mature oligodendrocytes (Dietrich et al., 2006). Our platform likely detected the acute cytoskeletal collapse associated with this direct effect. In contrast, tacrolimus-induced leukoencephalopathy is primarily driven by vascular endothelial dysfunction (PRES) (Hinchey et al., 1996), and white matter damage induced by methotrexate and 5-FU often involves microglial activation (Gibson et al., 2019) or delayed myelin degeneration (Han et al., 2008). These indirect mechanisms involving vascular or immune components, as well as long-term degenerative processes, are difficult to capture in a short-term monoculture assay. Therefore, this system may serve as a tool for screening compounds with direct cytotoxicity to oligodendrocytes, thereby addressing a critical gap in current safety evaluation protocols. Specifically, since the assay relies on the alignment of CLDN11 and MBP, it is particularly sensitive to compounds affecting cytoskeletal remodeling. Given that cytoskeletal reorganization is an essential prerequisite for process extension and initial ensheathment (Nawaz et al., 2015), this system provides a specific readout for this step.

Limitations of this study

This study has some limitations. First, while TEM analysis confirmed that oligodendrocytes physically wrapped around the nanofibers, the formation of compact multilayered myelin sheaths with high electron density was not observed. The formation of compact myelin may require longer culture periods or additional biological signals provided by neurons and astrocytes. Therefore, our platform should be interpreted as a model that evaluates the initiation and ensheathment efficacy of the myelination process rather than the completed compact myelin structure. However, given that ensheathment is the essential step for compaction and supports axonal metabolism, identifying compounds that promote this phase is highly relevant for drug discovery. Second, the evaluation of CLDN11 expression was limited to oligodendrocytes alone, and we did not consider the effects of neuronal function or interactions with other glial cells. Neuronal activity and astrocytic interactions promote myelin formation (Ishibashi et al., 2006; Wake et al., 2011). Considering these interactions, it is necessary to establish physiological models of neurons and other glial cells. Third, in the current study, we evaluated the ensheathment phase based on the expression of CLDN11 and morphological changes. This is a critical first step in assessing structural organization in drug screening. However, it remains unclear whether the formed ensheathment structures contribute to electrophysiological functional improvements, such as increased nerve conduction velocity. A key future task will be to apply this platform to a co-culture system with neurons to evaluate the insulating function of the formed myelin. Fourth, it is important to note that the upregulation of specific pathways observed in RNA-seq, such as ECM organization, was based on a modest fold-change threshold and has not yet been validated at the protein level. Finally, iPSCs derived from healthy individuals were used in this study. To model specific pathologies, such as MS or leukodystrophy, future studies should use patient-derived iPSCs or introduce inflammatory cytokines to mimic the disease microenvironment.

In conclusion, we successfully established a well-defined human oligodendrocyte differentiation system using TetON technology and nanofiber culture. Although the system models the ensheathment phase rather than myelin compaction, the quantitative evaluation of structural organization via CLDN11 expression and the transcriptomic insights into pro-myelinating molecular state provide a robust tool for evaluating compounds that modulate the initial phase of sheath formation. This platform provides a valuable tool for studying the pathophysiology of oligodendrocyte-related disorders.

RESOURCE AVAILABILITY

Lead contact

Further information and requests for resources and reagents should be directed to and will be fulfilled by Dr. Haruhisa Inoue (haruhisa@cira.kyoto-u.ac.jp).

Materials availability

The materials used in this study were commercially available. No new reagents were generated in this study.

Data and code availability

- The RNA-seq data generated in this study have been deposited in the Gene Expression Omnibus: GSE327118. The microscopy data reported in this manuscript will be shared with the lead contact upon request.
- This manuscript does not report original code.
- Any additional information required to reanalyze the data reported in this paper is available from the lead contact upon request.

ACKNOWLEDGMENTS

We thank all of our coworkers and collaborators for their kind support. We acknowledge Makiko Yasui, Rumi Ono, and Akiko Kadotani for their administrative assistance. This work was supported by the Japan Agency for Medical Research and Development under grant numbers JP23bm1323001, JP24fm0208101, JP23bm1423014, JP23bm1223013, JP23dk0207066, JPMH24FC 1008, JP24bm1123047, and JP24wm0625201 to H.I.; JP23bm 1423012 and JP24wm0625501 to T.K. and H.I.; Canon Foundation to H.I.

AUTHOR CONTRIBUTIONS

S.M., T.K., and H.I. conceived the project; S.M., Y.S., and K.T. performed the experiments; S.M., T.K., K.I., H.T., Y.K., T.I., Y.N., and H.I analyzed the data and provided scientific discussions; S.M., T.K., and H.I. wrote the manuscript.

DECLARATION OF INTERESTS

T.K., K.I., Y.S., K.T., and H.I. declare no competing interests. S.M., H.T., Y.K., T.I., and Y.N. are employees of Suntory Wellness Ltd., which markets health food products.

STAR★METHODS

Detailed methods are provided in the online version of this paper and include the following:

- KEY RESOURCES TABLE
- EXPERIMENTAL MODEL AND STUDY PARTICIPANTS DETAILS
  - Human iPSC maintenance
- METHOD DETAILS
  - Generation of iOligo from human iPSCs
  - Differentiation of NPCs from human iPSCs
  - Differentiation of oligodendrocytes

- ○ Neuron-oligodendrocyte co-culture
- ○ Compound treatment in cultured cells
- ○ Immunocytochemistry (ICC)
- ○ Time-lapse imaging
- ○ Quantitative RT-PCR
- ○ RNA sequencing
- ○ Transmission electron microscopy (TEM)
- ● QUANTIFICATION AND STATISTICAL ANALYSIS
  - ○ Statistical analysis

SUPPLEMENTAL INFORMATION

Supplemental information can be found online at https://doi.org/10.1016/j.stemcr.2026.102923.

Received: September 18, 2025
Revised: April 12, 2026
Accepted: April 13, 2026
Published: May 14, 2026

REFERENCES

Bercury, K.K., and Macklin, W.B. (2015). Dynamics and mechanisms of CNS myelination. Dev. Cell *32*, 447–458. https://doi.org/10.1016/j.devcel.2015.01.016.

Bernardo, A., Giammarco, M.L., De Nuccio, C., Ajmone-Cat, M.A., Visentin, S., De Simone, R., and Minghetti, L. (2017). Docosahexaenoic acid promotes oligodendrocyte differentiation via PPAR-γ signalling and prevents tumor necrosis factor-α-dependent maturational arrest. Biochim. Biophys. Acta. Mol. Cell Biol. Lipids *1862*, 1013–1023. https://doi.org/10.1016/j.bbalip.2017.06.014.

Buttery, P.C., and ffrench-Constant, C. (1999). Laminin-2/Integrin Interactions Enhance Myelin Membrane Formation by Oligodendrocytes. Mol. Cell. Neurosci. *14*, 199–212. https://doi.org/10.1006/mcne.1999.0781.

de Faria, O., Pivonkova, H., Varga, B., Timmler, S., Evans, K.A., and Káradóttir, R.T. (2021). Periods of synchronized myelin changes shape brain function and plasticity. Nat. Neurosci. *24*, 1508–1521. https://doi.org/10.1038/s41593-021-00917-2.

Depp, C., Sun, T., Sasmita, A.O., Spieth, L., Berghoff, S.A., Nazarenko, T., Overhoff, K., Steixner-Kumar, A.A., Subramanian, S., Arinrad, S., et al. (2023). Myelin dysfunction drives amyloid-β deposition in models of Alzheimer's disease. Nature *618*, 349–357. https://doi.org/10.1038/s41586-023-06120-6.

Devaux, J., and Gow, A. (2008). Tight junctions potentiate the insulative properties of small CNS myelinated axons. J. Cell Biol. *183*, 909–921. https://doi.org/10.1083/jcb.200808034.

Dietrich, J., Han, R., Yang, Y., Mayer-Pröschel, M., and Noble, M. (2006). CNS progenitor cells and oligodendrocytes are targets of chemotherapeutic agents in vitro and in vivo. J. Biol. *5*, 22. https://doi.org/10.1186/jbiol50.

Ehrlich, M., Mozafari, S., Glatza, M., Starost, L., Velychko, S., Hallmann, A.-L., Cui, Q.-L., Schambach, A., Kim, K.-P., Bachelin, C., et al. (2017). Rapid and efficient generation of oligodendrocytes from human induced pluripotent stem cells using transcription factors. Proc. Natl. Acad. Sci. USA *114*, E2243–E2252. https://doi.org/10.1073/pnas.1614412114.

García-León, J.A., Kumar, M., Boon, R., Chau, D., One, J., Wolfs, E., Eggermont, K., Berckmans, P., Gunhanlar, N., de Vrij, F., et al. (2018). SOX10 single transcription factor-based fast and efficient generation of oligodendrocytes from human pluripotent stem cells. Stem Cell Rep. *10*, 655–672. https://doi.org/10.1016/j.stemcr.2017.12.014.

Gargareta, V.-I., Reuschenbach, J., Siems, S.B., Sun, T., Piepkorn, L., Mangana, C., Späte, E., Goebbels, S., Huitinga, I., Möbius, W., et al. (2022). Conservation and divergence of myelin proteome and oligodendrocyte transcriptome profiles between humans and mice. eLife *11*, e77019. https://doi.org/10.7554/eLife.77019.

Gencic, S., Abuelo, D., Ambler, M., and Hudson, L.D. (1989). Pelizaeus-Merzbacher disease: an X-linked neurologic disorder of myelin metabolism with a novel mutation in the gene encoding proteolipid protein. Am. J. Hum. Genet. *45*, 435–442.

Gibson, E.M., Nagaraja, S., Ocampo, A., Tam, L.T., Wood, L.S., Pallegar, P.N., Greene, J.J., Geraghty, A.C., Goldstein, A.K., Ni, L., et al. (2019). Methotrexate chemotherapy induces persistent tri-glial dysregulation that underlies chemotherapy-related cognitive impairment. Cell *176*, 43–55.e13. https://doi.org/10.1016/j.cell.2018.10.049.

Gjervan, S.C., Ozgoren, O.K., Gow, A., Stockler-Ipsiroglu, S., and Pouladi, M.A. (2023). Claudin-11 in health and disease: implications for myelin disorders, hearing, and fertility. Front. Cell. Neurosci. *17*, 1344090. https://doi.org/10.3389/fncel.2023.1344090.

Gong, Z., Bilgel, M., Kiely, M., Triebswetter, C., Ferrucci, L., Resnick, S.M., Spencer, R.G., and Bouhrara, M. (2023). Lower myelin content is associated with more rapid cognitive decline among cognitively unimpaired individuals. Alzheimers Dement. *19*, 3098–3107. https://doi.org/10.1002/alz.12968.

Gow, A., Southwood, C.M., Li, J.S., Pariali, M., Riordan, G.P., Brodie, S.E., Danias, J., Bronstein, J.M., Kachar, B., and Lazzarini, R.A. (1999). CNS myelin and sertoli cell tight junction strands are absent in Osp/Claudin-11 null mice. Cell *99*, 649–659. https://doi.org/10.1016/S0092-8674(00)81553-6.

Han, R., Yang, Y.M., Dietrich, J., Luebke, A., Mayer-Pröschel, M., and Noble, M. (2008). Systemic 5-fluorouracil treatment causes a syndrome of delayed myelin destruction in the central nervous system. J. Biol. *7*, 12. https://doi.org/10.1186/jbiol69.

Hasegawa, M., Houdou, S., Mito, T., Takashima, S., Asanuma, K., and Ohno, T. (1992). Development of myelination in the human fetal and infant cerebrum: a myelin basic protein immunohistochemical study. Brain Dev. *14*, 1–6. https://doi.org/10.1016/S0387-7604(12)80271-3.

Hinchey, J., Chaves, C., Appignani, B., Breen, J., Pao, L., Wang, A., Pessin, M.S., Lamy, C., Mas, J.-L., and Caplan, L.R. (1996). A reversible posterior leukoencephalopathy syndrome. N. Engl. J. Med. *334*, 494–500. https://doi.org/10.1056/NEJM199602223340803.

Inoue, H., Nagata, N., Kurokawa, H., and Yamanaka, S. (2014). iPS cells: A game changer for future medicine. EMBO J. *33*, 409–417. https://doi.org/10.1002/embj.201387098.

Ishibashi, T., Dakin, K.A., Stevens, B., Lee, P.R., Kozlov, S.V., Stewart, C.L., and Fields, R.D. (2006). Astrocytes promote myelination in response to electrical impulses. Neuron *49*, 823–832. https://doi.org/10.1016/j.neuron.2006.02.006.

Jäkel, S., Agirre, E., Mendanha Falcão, A., van Bruggen, D., Lee, K.W., Knuesel, I., Malhotra, D., ffrench-Constant, C., Williams, A., and Castelo-Branco, G. (2019). Altered human oligodendrocyte heterogeneity in multiple sclerosis. Nature *566*, 543–547. https://doi.org/10.1038/s41586-019-0903-2.

Jorstad, N.L., Song, J.H.T., Exposito-Alonso, D., Suresh, H., Castro-Pacheco, N., Krienen, F.M., Yanny, A.M., Close, J., Gelfand, E., Long, B., et al. (2023). Comparative transcriptomics reveals human-specific cortical features. Science *382*, eade9516. https://doi.org/10.1126/science.ade9516.

Kim, S.-I., Oceguera-Yanez, F., Sakurai, C., Nakagawa, M., Yamanaka, S., and Woltjen, K. (2015). Inducible transgene expression in human iPS cells using versatile All-in-One piggyBac transposons. In Methods in Molecular Biology (Humana Press), pp. 111–131.

Kondo, T., Imamura, K., Funayama, M., Tsukita, K., Miyake, M., Ohta, A., Woltjen, K., Nakagawa, M., Asada, T., Arai, T., et al. (2017). iPSC-based compound screening and in vitro trials identify a synergistic anti-amyloid β combination for alzheimer's disease. Cell Rep. *21*, 2304–2312. https://doi.org/10.1016/j.celrep.2017.10.109.

Lee, S., Leach, M.K., Redmond, S.A., Chong, S.Y.C., Mellon, S.H., Tuck, S.J., Feng, Z.-Q., Corey, J.M., and Chan, J.R. (2012). A culture system to study oligodendrocyte myelination processes using engineered nanofibers. Nat. Methods *9*, 917–922. https://doi.org/10.1038/nmeth.2105.

Lubetzki, C., Zalc, B., Williams, A., Stadelmann, C., and Stankoff, B. (2020). Remyelination in multiple sclerosis: from basic science to clinical translation. Lancet Neurol. *19*, 678–688. https://doi.org/10.1016/S1474-4422(20)30140-X.

Madhavan, M., Nevin, Z.S., Shick, H.E., Garrison, E., Clarkson-Paredes, C., Karl, M., Clayton, B.L.L., Factor, D.C., Allan, K.C., Barbar, L., et al. (2018). Induction of myelinating oligodendrocytes in human cortical spheroids. Nat. Methods *15*, 700–706. https://doi.org/10.1038/s41592-018-0081-4.

Morita, K., Sasaki, H., Fujimoto, K., Furuse, M., and Tsukita, S. (1999). Claudin-11/OSP-based tight junctions of myelin sheaths in brain and sertoli cells in testis. J. Cell Biol. *145*, 579–588. https://doi.org/10.1083/jcb.145.3.579.

Nakagawa, M., Taniguchi, Y., Senda, S., Takizawa, N., Ichisaka, T., Asano, K., Morizane, A., Doi, D., Takahashi, J., Nishizawa, M., et al. (2014). A novel efficient feeder-free culture system for the derivation of human induced pluripotent stem cells. Sci. Rep. *4*, 3594. https://doi.org/10.1038/srep03594.

Nakano, S., Uyeda, A., Matsunaga, Y.T., and Muramatsu, R. (2023). Phenotypic and transcriptional characterization of oligodendrocyte precursor cells in a 3D culture. Biomater. Sci. *11*, 2860–2869. https://doi.org/10.1039/d2bm01897g.

Nasrabady, S.E., Rizvi, B., Goldman, J.E., and Brickman, A.M. (2018). White matter changes in Alzheimer's disease: a focus on myelin and oligodendrocytes. Acta Neuropathol. Commun. *6*, 22. https://doi.org/10.1186/s40478-018-0515-3.

Nave, K.-A., and Werner, H.B. (2021). Ensheathment and myelination of axons: evolution of glial functions. Annu. Rev. Neurosci. *44*, 197–219. https://doi.org/10.1146/annurev-neuro-100120-122621.

Nawaz, S., Sánchez, P., Schmitt, S., Snaidero, N., Mitkovski, M., Velte, C., Brückner, B.R., Alexopoulos, I., Czopka, T., Jung, S.Y., et al. (2015). Actin filament turnover drives leading edge growth during myelin sheath formation in the central nervous system. Dev. Cell *34*, 139–151. https://doi.org/10.1016/j.devcel.2015.05.013.

Neman, J., and de Vellis, J. (2008). Myelinating cells in the central nervous system—development, aging, and disease. In Handbook of Neurochemistry and Molecular Neurobiology, A. Lajtha, J.R. Perez-Polo, and S. Rossner, eds. (Springer), pp. 61–75.

Ng, A.H.M., Khoshakhlagh, P., Rojo Arias, J.E., Pasquini, G., Wang, K., Swiersy, A., Shipman, S.L., Appleton, E., Kiaee, K., Kohman, R.E., et al. (2021). A comprehensive library of human transcription factors for cell fate engineering. Nat. Biotechnol. *39*, 510–519. https://doi.org/10.1038/s41587-020-0742-6.

O'Meara, R.W., Michalski, J.-P., and Kothary, R. (2011). Integrin signaling in oligodendrocytes and its importance in CNS myelination. J. Signal Transduct. *2011*, 354091. https://doi.org/10.1155/2011/354091.

Ransohoff, R.M. (2012). Animal models of multiple sclerosis: the good, the bad and the bottom line. Nat. Neurosci. *15*, 1074–1077. https://doi.org/10.1038/nn.3168.

Ransom, B.R., Goldberg, M.P., Arai, K., and Baltan, S. (2021). White matter pathophysiology. In Stroke: Pathophysiology, Diagnosis, and Management (Elsevier), pp. 103–116.e4.

Riedhammer, K.M., Stockler, S., Ploski, R., Wenzel, M., Adis-Dutschmann, B., Ahting, U., Alhaddad, B., Blaschek, A., Haack, T.B., Kopajtich, R., et al. (2021). De novo stop-loss variants in CLDN11 cause hypomyelinating leukodystrophy. Brain *144*, 411–419. https://doi.org/10.1093/brain/awaa410.

Schepers, M., Malheiro, A., Gamardo, A.S., Hellings, N., Prickaerts, J., Moroni, L., Vanmierlo, T., and Wieringa, P. (2023). Phosphodiesterase (PDE) 4 inhibition boosts Schwann cell myelination in a 3D regeneration model. Eur. J. Pharm. Sci. *185*, 106441. https://doi.org/10.1016/j.ejps.2023.106441.

Sherman, D.L., and Brophy, P.J. (2005). Mechanisms of axon ensheathment and myelin growth. Nat. Rev. Neurosci. *6*, 683–690. https://doi.org/10.1038/nrn1743.

Takagi, D., Tsukamoto, S., Nakade, K., Shimizu, T., Arai, Y., Matsuo-Takasaki, M., Noguchi, M., Nakamura, Y., Yumoto, N., Kawada, J., et al. (2024). Generation of MBP-tdTomato reporter human induced pluripotent stem cell line for live myelin visualization. Stem Cell Res. *79*, 103493. https://doi.org/10.1016/j.scr.2024.103493.

Trapp, B.D., and Bernsohn, J. (1978). Essential fatty acid deficiency and CNS myelin. J. Neurol. Sci. *37*, 249–266. https://doi.org/10.1016/0022-510X(78)90207-1.

Uchida, Y., Sumiya, T., Tachikawa, M., Yamakawa, T., Murata, S., Yagi, Y., Sato, K., Stephan, A., Ito, K., Ohtsuki, S., et al. (2019). Involvement of Claudin-11 in disruption of blood-brain, -spinal cord, and -arachnoid barriers in multiple sclerosis. Mol. Neurobiol. *56*, 2039–2056. https://doi.org/10.1007/s12035-018-1207-5.

von der Bey, M., De Cicco, S., Zach, S., Hengerer, B., and Ercan-Herbst, E. (2023). Three-dimensional co-culture platform of human induced pluripotent stem cell-derived oligodendrocyte lineage cells and neurons for studying myelination. STAR Protoc. *4*, 102164. https://doi.org/10.1016/j.xpro.2023.102164.

Wake, H., Lee, P.R., and Fields, R.D. (2011). Control of local protein synthesis and initial events in myelination by action potentials. Science *333*, 1647–1651. https://doi.org/10.1126/science.1206998.

Yang, M., Martin, C.J.L., Kowsari, K., Jagielska, A., and Van Vliet, K.J. (2025). Myelin ensheathment and drug responses of oligodendrocytes are modulated by stiffness of artificial axons. PLoS One *20*, e0290521. https://doi.org/10.1371/journal.pone.0290521.

Zeiss, C.J. (2021). Comparative milestones in rodent and human postnatal central nervous system development. Toxicol. Pathol. *49*, 1368–1373. https://doi.org/10.1177/01926233211046933.

STAR★METHODS

KEY RESOURCES TABLE

REAGENT or RESOURCE	SOURCE	IDENTIFIER
**Antibodies**		
Rat monoclonal anti-MBP	Merck	Cat# MAB386, RRID:AB_94975
Rabbit polyclonal anti-Claudin-11 antibody	Thermo Fisher	Cat# 36–4500, RRID:AB_2533259
Rabbit polyclonal anti-Neurofilament L antibody	Merck	Cat# AB9568, RRID:AB_11213875
Mouse monoclonal anti-Integrin beta 1 antibody	Abcam	Cat# ab30394, RRID:AB_775726
Goat anti-Mouse, Alexa Fluor 488	Thermo Fisher	Cat# A-32723, RRID:AB_2633275
Goat anti-Rabbit, Alexa Fluor 546	Thermo Fisher	Cat# A-11010, RRID:AB_2534077
Goat anti-Rat, Alexa Fluor 647	Thermo Fisher	Cat# A-21247, RRID:AB_141778
**Chemicals, peptides, and recombinant proteins**		
StemFit AK02N	Ajinomoto	Cat# RCAK02N
iMatrix-511	Nippi	Cat# 892012
DMEM/F-12	Thermo Fisher	Cat# 11320033
Neurobasal™ Medium	Thermo Fisher	Cat# 21103049
Neurobasal™ Plus Medium	Thermo Fisher	Cat# A3582901
B-27™ Plus Supplement	Thermo Fisher	Cat# A3582801
B-27™ Supplement, minus vitamin A	Thermo Fisher	Cat# 12587010
N-2 Supplement	Thermo Fisher	Cat# 17502048
KnockOut Serum Replacement (KSR)	Thermo Fisher	Cat# 10828028
GlutaMAX	Thermo Fisher	Cat# 35050-061
Matrigel	Corning	Cat# 354234
Synthemax II-SC	Corning	Cat# 3535
CellNest	FujiFilm	Cat# 16461438
Poly-L-lysine	Merck	Cat# P4707
Poly-*l*-lysine-FITC Labeled	Merck	Cat# P3543
Doxycyclin	Takara	Cat# 631311
G418 Disulfate	Nacalai Tesque	Cat# 09380-86
Zeocin	Nacalai Tesque	Cat# 61483-26
MEM Non-Essential Amino Acids Solution	Thermo Fisher	Cat# 11140050
TrypLE™ Select Enzyme	Thermo Fisher	Cat# 12563011
2-Mercaptoethanol	Thermo Fisher	Cat# 21985023
BDNF	Thermo Fisher	Cat# 450-02

(*Continued on next page*)

Continued

REAGENT or RESOURCE	SOURCE	IDENTIFIER
GDNF	Thermo Fisher	Cat# 450-10
NT-3	Thermo Fisher	Cat# 450-03
LIF	Thermo Fisher	Cat# 300-05
PDGF-AA	Thermo Fisher	Cat# 100-13A
IGF-I	Thermo Fisher	Cat# 100-11
SB431542	Cayman Chemical	Cat# 13031
Dorsomorphin	Merck	Cat# P5499
Y-27632	Nacalai Tesque	Cat# 18188-04
Purmorphamine	Selleck	Cat# S3042
Ascorbic acid	Merck	Cat# A4544
3, 3′, 5-Triiodo-L-thyronine Sodium Salt (T3)	FujiFilm	Cat# 038-25541
SAG	Enzo	Cat# ALX-270-426-M001
CHIR-99021	Cayman	Cat# 13122
db-cAMP	Merck	Cat# D0627
Cytochalasin D	Selleck	Cat# S8184
Roflumilast	Selleck	Cat# S2131
cis-4,7,10,13,16,19-docosahexaenoic acid	Merck	Cat# D2534
Arachidonic acid	Merck	Cat# A3611
Tacrolimus Monohydrate	TCI	Cat# M2258
Cytarabine	TCI	Cat# C2035
cis-Diammineplatinum(II) Dichloride	TCI	Cat# D3371
5-Fluorouracil	TCI	Cat# F0151
Methotrexate Hydrate	TCI	Cat# M1664
Normal Goat Serum	Abcam	Cat# ab7481
DAPI	Thermo Fisher	Cat# 62248
Stem-Cellbanker	TAKARA	Cat# CB047
**Critical commercial assays**		
RNeasy Plus Mini kit	QIAGEN	Cat# 74136
High capacity cDNA Reverse Transcription kit with Rnase inhibitor	Thermo Fisher	Cat# 4374966
TaqMan Fast Universal PCR Master Mix	Thermo Fisher	Cat# 2654715
Lipofectamine™ LTX Reagent with PLUS™ Reagent	Thermo Fisher	Cat# 15338100
NanoAligned, 96 Well Plate	Nanofiber Solutions	Cat# 9602
**Deposited data**		
RNA-seq, raw, and analyzed data	This paper	GEO: GSE327118

(*Continued on next page*)

Continued		
**REAGENT or RESOURCE**	**SOURCE**	**IDENTIFIER**
**Experimental models: Cell lines**		
Human iPSC	RIKEN BioResource Research Center	201B7
Human iPSC	RIKEN BioResource Research Center	HPS1748
Human iPSC	RIKEN BioResource Research Center	HPS5652
Human iN-iPSC	(Kondo et al., 2017)	201B7
**Oligonucleotides**		
SOX10	DANAFORM	Cat# 100000537
OLIG2	Kazusa	Cat# FXC27157
NKX6-2	Horizon Discovery	Cat# MHS6278-202857555
ACTB	Thermo Fisher	Cat# Hs99999903_m1
SOX10	Thermo Fisher	Cat# Hs00366918_m1
OLIG2	Thermo Fisher	Cat# Hs00300164_s1
NKX6-2	Thermo Fisher	Cat# Hs00752986_s1
MBP	Thermo Fisher	Cat# Hs00921945_m1
PDGFRA	Thermo Fisher	Cat# Hs00998018_m1
MYRF	Thermo Fisher	Cat# Hs00973739_m1
PLP1	Thermo Fisher	Cat# Hs00166914_m1
PMP2	Thermo Fisher	Cat# Hs00160204_m1
CLDN11	Thermo Fisher	Cat# Hs00194440_m1
**Software and algorithms**		
SPSS	IBM	Version 26
Prism Software	Graphpad	Version 10.4
ImageJ	NIH	Version 1.54f
R	R Foundation for Statistical Computing	Version 4.4.2

EXPERIMENTAL MODEL AND STUDY PARTICIPANTS DETAILS

Human iPSC maintenance

The data on human iPSCs used in the present study are shown in Table S2 and key resources table. The use of iPSCs was approved by the Ethics Committee of the RIKEN BioResource Research Center. Human iPSCs were provided by the RIKEN BioResource Research Center through the National BioResource Project of MEXT (Japan). iPSCs were maintained on laminin (iMatrix-511; TAKARA, Kusatsu, Japan) in StemFit AK02N (Ajinomoto, Tokyo, Japan) at 37°C in a 5% CO_2 incubator as previously described (Nakagawa et al., 2014), with slight modifications. iOligo-iPSCs or iN-iPSCs were maintained in StemFit AK02N containing 50 μg/mL G418 disulfate (Nacalai Tesque, Kyoto, Japan) or 0.2 μg/mL zeocin (Nacalai Tesque). Passages were performed every seventh day. Cells were dissociated using TrypLE select enzyme (Thermo Fisher Scientific Inc., Waltham, MA, USA) and cultured in StemFit AK02N with 10 μM Y-27632 (Nacalai Tesque). The medium was changed to remove Y-27632 on the following day. iPSCs were cryopreserved using Stem-Cellbanker (TAKARA) to prepare frozen stocks containing 2×10^5 cells per vial. The number of subsequent passages during experimentation was limited to approximately 10 passages. iPSCs in culture were confirmed to be mycoplasma-negative every three months, and to express the pluripotency markers NANOG and TRA1-60.

METHOD DETAILS

Generation of iOligo from human iPSCs

Direct conversion technology was utilized to establish robust and rapid oligodendrocyte differentiation. Human Sox10, Olig2, and Nkx6.2 cDNA, under the tetracycline-inducible promoter (tetO), was transfected into iPSCs using a PiggyBac transposon system (Kim et al., 2015) and Lipofectamine LTX with Plus Reagent (Thermo Fisher). A vector containing tetO: Sox10, Olig2, Nkx6.2 was used (Figure 1A). After the antibiotic selection of G418 disulfate (Nacalai Tesque), colonies and subclones that could efficiently differentiate into oligodendrocytes by inducing the temporal expression of Sox10, Olig2, and Nkx6.2 were selected.

Differentiation of NPCs from human iPSCs

The iOligo-iPSCs were dissociated using TrypLE Select enzymes and placed in a U-bottom 96-well plates (Thermo Fisher) at densities of 2.0×10^4 cells/well to form embryonic bodies (EBs) for suspension culture. From day 0 to day 2, EBs were cultured in DMEM/F12 (Thermo Fisher) containing 20% KSR (Thermo Fisher), NEAA (Thermo Fisher), GlutaMAX (Thermo Fisher), 0.1 mM 2-mercaptoethanol (Thermo Fisher), antibiotics with 10 μM Y-27632, 3 μM CHIR99021 (Merck), 0.5 μM purmorphamine (Selleck), 1 μM dorsomorphin (Thermo Fisher), and 10 μM SB431542 (Thermo Fisher). On day 2, the medium was changed to N2B27 medium consisting of equal parts DMEM/F12 and Neurobasal (Thermo Fisher) with 0.5% N2 supplement (Thermo Fisher), 0.5% B27 supplement lacking vitamin A (Thermo Fisher), GlutaMAX, antibiotics, with the same concentrations of CHIR99021, purmorphamine, dorsomorphin, and SB431542. On day 5, the medium was changed to N2B27 medium with CHIR99021, purmorphamine, and 150 μM ascorbic acid (Merck). On day 7, EBs were dissociated by TrypLE and plated on Matrigel-coated (Corning) 6-well plates in medium consisting of N2B27 medium supplemented with 3 μM CHIR99021, 0.5 μM SAG (Merck), and 150 μM ascorbic acid. Cells were maintained in N2B27 medium with CHIR99021, SAG, and ascorbic acid and passaged twice weekly. From day 19 onwards, the cells were used to induce oligodendrocyte differentiation.

Differentiation of oligodendrocytes

NPCs were dissociated by TrypLE Select. For conventional culture, NPCs were transferred to tissue culture plates (Corning) coated with Matrigel (1:30 dilution) for 2 h at 37°C. For differentiation on nanofibers, 24-well nanofiber inserts or 96-well nanofiber plates (Nanofiber Solutions, Dublin, OH, USA) were coated with Matrigel (1:30 dilution) and laminin (1.7 μg/mL) for 2 h at 37°C. In experiments requiring nanofiber visualization, this coating was supplemented with FITC-labeled poly-L-lysine (0.1 mg/mL). NPCs were seeded at densities of 2.5×10^5 cells/well for a 24-well scale or 5.0×10^4 cells/well for a 96-well scale. On day 0, cells were cultured in DMEM/F12 containing 0.5% N2 supplement, 0.5% B27 supplement lacking vitamin A, GlutaMAX, antibiotics, 1 μM SAG, 200 μM ascorbic acid, 10 ng/mL IGF-I (Thermo Fisher), 10 ng/mL PDGF-AA (Thermo Fisher), 10 ng/mL 3, 3′, 5-triiodo-L-thyronine sodium salt (T3) (FujiFilm), 0.1% trace elements cocktail solution (0.17 ng/mL $MnSO_4 \cdot H_2O$, 140 ng/mL $Na_2SiO_3 \cdot 9H_2O$, 1.24 ng/mL molybdic acid ammonium salt, 0.65 ng/mL NH_4VO_3, 0.13 ng/mL $NiSO_4 \cdot 6H_2O$, 0.12 ng/mL $SnCI_2$), 10 μM Y-27632, and 2 μg/mL doxycycline (Takara). On day 4, the medium was changed to DMEM/F12 containing 0.5% N2 supplement, 0.5% B27 supplement lacking vitamin A, GlutaMAX, antibiotics, 200 μM ascorbic acid, 100 μM db-cAMP (Merck), 10 ng/mL IGF-I, 60 ng/mL T3, 0.1% trace elements cocktail solution, and 2 μg/mL doxycycline. For the initial characterization of differentiation efficiency and robustness (Figures 1 and S1; Table S1), cells were analyzed one week after doxycycline induction. For neuron-oligodendrocyte co-culture, nanofiber wrapping, and compound assays (Figures 2, 3, 4, and 5), cells were cultured for two weeks after doxycycline induction to ensure sufficient morphological maturation. From day 7 onwards, the medium was changed to the differentiation medium consisting of N2B27 medium supplemented with 200 μM ascorbic acid, 100 μM db-cAMP (Merck), 10 ng/mL IGF-I, 60 ng/mL T3, 0.1% trace elements cocktail solution, 10 ng/mL BDNF (Thermo Fisher), 10 ng/mL GDNF (Thermo Fisher), 10 ng/mL NT-3 (Thermo Fisher), 1 ng/mL LIF (Thermo Fisher), and 2 μg/mL doxycycline for maturation. The culture medium was changed twice weekly.

Neuron-oligodendrocyte co-culture

Human neurons used for neuron–oligodendrocyte co-culture were differentiated using direct conversion technology, as previously described (Kondo et al., 2017). On day 0, iN-iPSCs were dissociated with TrypLE Select and seeded onto 6-well plates coated with a mixture of poly-L-lysine (0.0002% w/v; Merck, Darmstadt, Germany), Matrigel (2% v/v; Corning), Synthemax 2-SC (20 μg/mL; Corning), and CellNest (2% v/v; FujiFilm, Tokyo, Japan) at densities of 3.0×10^6 cells/well. The cells were

cultured in Neurobasal plus medium containing 2% B-27 plus supplement (Thermo Fisher), 2 μg/mL doxycycline (FujiFilm), 10 μM Y-27632, and antibiotics. On day 5, differentiated neurons were dissociated, placed at densities of 5.0×10^4 cells/well on differentiated oligodendrocytes which had been cultured with doxycycline for 1 week in 96-well plates. The neuron–oligodendrocyte co-cultures were maintained in differentiation medium with 10 μM Y-27632 for an additional week.

Compound treatment in cultured cells

Cytochalasin-D (Selleck), roflumilast (Selleck), DHA (Merck), ARA (Merck), cisplatin (TCI), 5-fluorouracil (TCI), methotrexate (TCI), and tacrolimus (TCI) were dissolved in dimethyl sulfoxide (Nacalai Tesque) and cytarabine (TCI) was dissolved in MilliQ. Compounds were added to the medium at a final concentration of 0.1% v/v. The culture medium was changed twice per week.

Immunocytochemistry (ICC)

Cells were fixed in 4% paraformaldehyde (PFA) (Nacalai Tesque) for 10 min at room temperature, permeabilized in 0.1% Triton X-100 (Nacalai Tesque) for 10 min at room temperature, and incubated with 5% goat serum (Abcam, Cambridge, UK). Incubation with primary antibodies was performed at 4°C overnight. After incubation with secondary antibodies for 1 h at room temperature, DAPI (Thermo Fisher) was incubated for 10 min at room temperature. Cell images were acquired using Operaphenix (PerkinElmer, Waltham, MA, USA) and analyzed using the Harmony software (PerkinElmer). The primary and secondary antibodies used in this assay are listed in key resources table. To quantify the proportion of MBP-positive cells, the total number of nuclei (DAPI-positive) and nuclei within the MBP-immunoreactive regions were counted. To analyze CLDN11 expression, the CLDN11 signal present within the identified MBP-positive regions was detected and its signal intensity was calculated. Similar analyses across the different acquired images were performed. The orientation of CLDN11 and nanofibers was quantified using the ImageJ OrientationJ plugin (NIH, Bethesda, MD, USA). Local orientation angles ranging from −90° to +90° were evaluated using the "Distribution" function based on the structure tensor method with a cubic spline gradient.

Time-lapse imaging

Time-lapse imaging was conducted using CellVoyager CV8000 (Yokogawa Electric, Tokyo, Japan) at 37°C, 5% CO_2. TdTomato-expressing cells were excited at 561 nm, from which fluorescence was obtained using a 600 nm channel. Images were captured every 30 min for 48 h. The acquired images were analyzed using ImageJ software, and videos were obtained from sequential images.

Quantitative RT-PCR

Total RNA was extracted using the RNeasy Plus mini kit (QIAGEN, Hilden, Germany). 500 ng of total RNA was reverse transcribed into cDNA using a High-Capacity cDNA Reverse Transcription kit with an RNase inhibitor (Thermo Fisher). The PCR primers used to analyze gene expression are listed in key resources table. The generated cDNA was used as a template for each reaction in a real-time quantitative PCR using StepOne Plus (Thermo Fisher). Actb was used as a housekeeping control gene to normalize the target genes. Relative quantification was performed using the comparative threshold (Ct) cycle method. Fold changes were calculated relative to cells cultured without doxycycline.

RNA sequencing

Total RNA was extracted using the RNeasy Plus mini kit (QIAGEN), and RNA integrity was assessed using an Agilent TapeStation (Agilent Technologies, Santa Clara, CA, USA). cDNA libraries were constructed using the SMART-Seq mRNA kit (TAKARA), followed by purification with AMPure XP beads (Beckman Coulter). Subsequently, fragmentation and tagging were performed using the Nextera XT DNA Library Prep Kit and DNA/RNA UD Indexes (Illumina) via transposon-based tagmentation. Sequencing was conducted on an Illumina NovaSeq X Plus system using the NovaSeq X Series 25B Reagent Kit in a 150-bp paired-end configuration. Raw BCL data were converted to FASTQ files using bcl2fastq2 v2.20 (Illumina). Read mapping and gene quantification were performed using the DRAGEN Bio-IT Platform v4.3.6 (Illumina) against the GRCh38 reference genome and annotation (GENCODE release 39). Statistical analyses were performed using R software (v4.4.2). Genes with low expression (total counts <10) were filtered out, and differentially expressed genes (DEGs) were identified using the DESeq2 package with a threshold of adjusted p-value <0.05 and $|\log2 \text{ fold change}| > 0.585$. Principal Component Analysis (PCA) was performed to visualize sample variance. Volcano plots and heatmaps were generated using EnhancedVolcano and pheatmap packages, respectively. Gene Ontology (GO) enrichment analysis was conducted using the clusterProfiler package. Functional clusters of GO terms were visualized as tree plots using

enrichplot package. The Benjamini-Hochberg (BH) method was used for multiple testing correction, with significant terms defined by an adjusted p-value <0.05.

Transmission electron microscopy (TEM)

Samples were fixed with 2% paraformaldehyde and 2% glutaraldehyde in 0.1 M phosphate buffer (pH 7.4), followed by overnight fixation in 2% glutaraldehyde at 4°C. After post-fixation with 2% osmium tetroxide for 1 h, specimens were dehydrated in a graded ethanol series and embedded in Quetol-812 resin (Nisshin EM Co., Tokyo, Japan). Ultrathin sections (70 nm) were prepared using an Ultracut UCT ultramicrotome (Leica, Vienna, Austria) and double-stained with 2% uranyl acetate and lead stain solution (Merck). Images were acquired using a JEM-1400Plus transmission electron microscope (JEOL Ltd., Tokyo, Japan) at an acceleration voltage of 100 kV equipped with a CCD camera (EM-14830RUBY2; JEOL Ltd.).

QUANTIFICATION AND STATISTICAL ANALYSIS

Statistical analysis

Data are expressed as mean ± standard error (SE). The results were analyzed using unpaired t test or Dunnett's test to determine the statistical significance of the data compared to the control. All analyses were performed using SPSS Statistics 26 (SPSS Inc., Chicago, IL, USA), R software version 4.4.2 (R Foundation for Statistical Computing), and Prism 10 (GraphPad Software, Boston, MA, USA).

Stem Cell Reports, Volume *21*

Supplemental Information

Nanofiber-based platform for quantitative analysis of human oligodendrocyte ensheathment with pharmacological perturbations

Satoshi Morita, Takayuki Kondo, Keiko Imamura, Yukako Sagara, Kayoko Tsukita, Hisanori Tokuda, Yoshihisa Kaneda, Takayuki Izumo, Yoshihiro Nakao, and Haruhisa Inoue

Figure S1

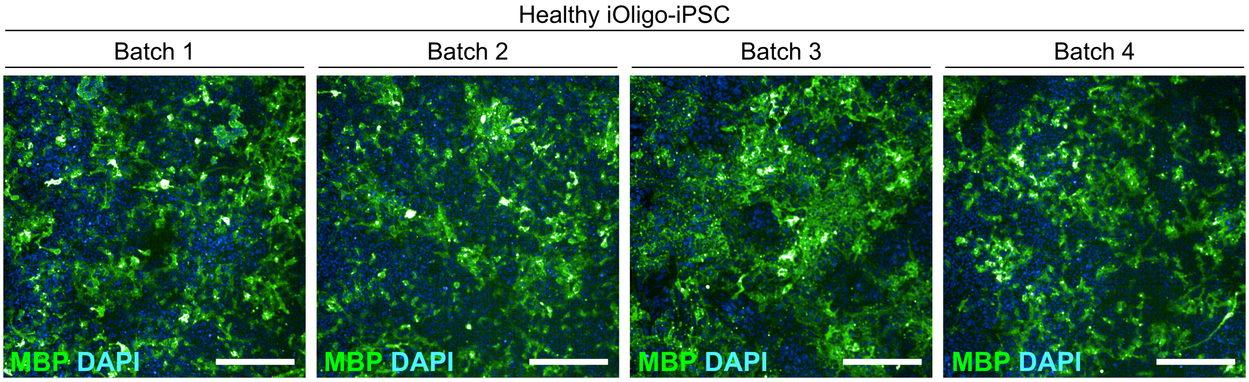

Healthy iOligo-iPSC
Batch 1
Batch 2
Batch 3
Batch 4
MBP DAPI
MBP DAPI
MBP DAPI
MBP DAPI

Figure S2

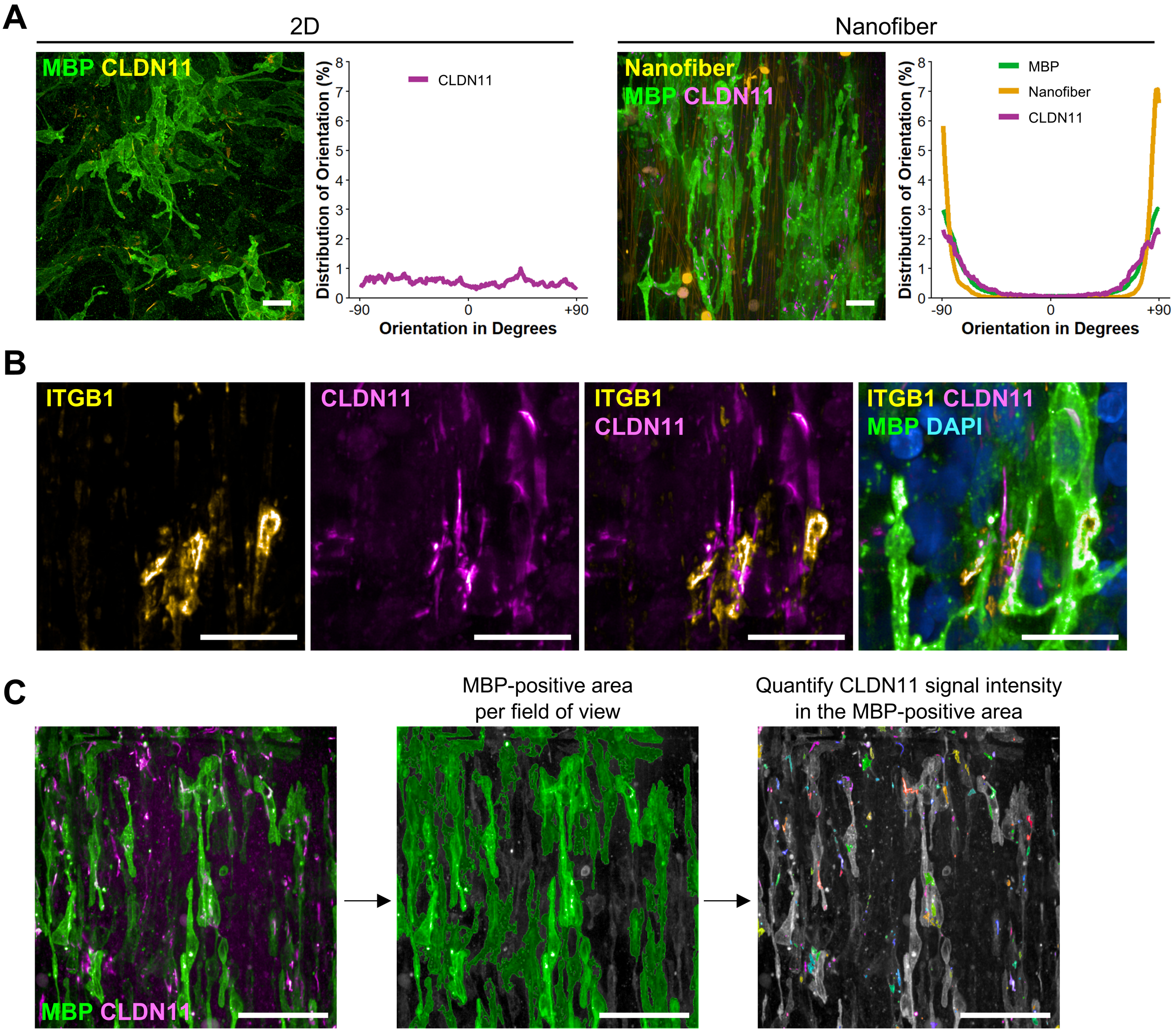
A
2D
Nanofiber
MBP CLDN11
CLDN11
Distribution of Orientation (%)
8
7
6
5
4
3
2
1
0
-90
0
+90
Orientation in Degrees
Nanofiber
MBP CLDN11
MBP
Nanofiber
CLDN11
B
ITGB1
CLDN11
ITGB1
CLDN11
ITGB1 CLDN11
MBP DAPI
C
MBP-positive area
per field of view
Quantify CLDN11 signal intensity
in the MBP-positive area
MBP CLDN11

Figure S3

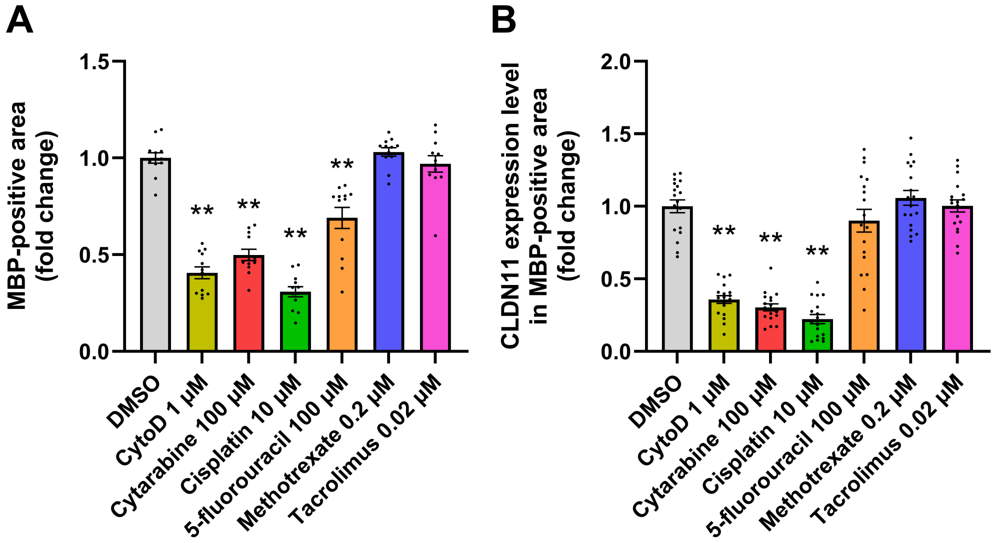
A
MBP-positive area (fold change)
1.5
1.0
0.5
0.0
**
**
**
**
DMSO
CytoD 1 μM
Cytarabine 100 μM
Cisplatin 10 μM
5-fluorouracil 100 μM
Methotrexate 0.2 μM
Tacrolimus 0.02 μM
B
CLDN11 expression level in MBP-positive area (fold change)
2.0
1.5
1.0
0.5
0.0
**
**
**
DMSO
CytoD 1 μM
Cytarabine 100 μM
Cisplatin 10 μM
5-fluorouracil 100 μM
Methotrexate 0.2 μM
Tacrolimus 0.02 μM

Supplemental figure legends

Figure S1. Differentiated oligodendrocytes across independent batches.

Representative images of 201B7-iOligo from four independent differentiation batches cultured for 1 week with doxycycline. Scale bars, 200 μm.

Figure S2. Analysis of CLDN11 expression relative to nanofibers.

(A) Representative images of CLDN11 expression in conventional 2D culture and on nanofibers. The orientation of CLDN11, MBP, and nanofibers was quantified using the ImageJ OrientationJ plugin. Local orientation angles ranging from -90° to +90° were evaluated using the “Distribution” function based on the structure tensor method with a cubic spline gradient. Histograms show the normalized relative frequency (%) of the orientation distribution. Scale bars, 20 μm.

(B) Representative images of ITGB1 and CLDN11 expression in cells cultured for two weeks on nanofibers. Scale bars, 20 μm.

(C) Analytical workflow for MBP-positive area detection and quantification of CLDN11 signal intensity. Scale bars, 50 μm.

Figure S3. Evaluation of drug-induced leukoencephalopathy agents.

The MBP-positive area and CLDN11 signal intensity within the MBP-positive area were calculated. Data represent mean ± SE. n = 12 (A) or 18 (B) images were analyzed per condition, obtained from 3 independent cultures. Statistical comparisons were performed using Dunnett’s test compared to DMSO control. $**p < 0.01$.

Table S1. The interbatch coefficient of variation, related to Figure 1.

Parameter	MBP-positive cells (%)
Batch 1	30.4
Batch 2	22.9
Batch 3	40.8
Batch 4	31.7
Mean ± SD	31.4 ± 7.3
CV (%)	23.2

Table S2. List of iPSCs used in this study

iPSCs	Age	Gender	Race	Disease
201B7	30s	Female	Caucasian	Healthy
HPS5652	30s	Male	Asian	Healthy
HPS1748	60s	Male	Japanese	Healthy
